# Revisiting Abnormalities of Ventricular Depolarization: Redefining Phenotypes and Associated Outcomes Using Tree‐Based Dimensionality Reduction

**DOI:** 10.1161/JAHA.124.040814

**Published:** 2025-06-18

**Authors:** Mehak Gurnani, Konstantinos Patlatzoglou, Joseph Barker, Derek Bivona, Libor Pastika, Ewa Sieliwonczyk, Boroumand Zeidaabadi, Paolo Inglese, Lara Curran, Ahran D. Arnold, Declan O'Regan, Zachary Whinnett, Kenneth C. Bilchick, Nicholas S. Peters, Daniel B. Kramer, Jonathan W. Waks, Arunashis Sau, Fu Siong Ng

**Affiliations:** ^1^ National Heart and Lung Institute, Imperial College London London UK; ^2^ Department of Biomedical Engineering University of Virginia Charlottesville VA USA; ^3^ MRC Laboratory of Medical Sciences Imperial College London London UK; ^4^ University of Antwerp and Antwerp University Hospital Antwerp Belgium; ^5^ Istituto Italiano di Tecnologia Genoa Italy; ^6^ Department of Cardiology Imperial College Healthcare NHS Trust London UK; ^7^ Department of Medicine University of Virginia Health System Charlottesville VA USA; ^8^ Richard A. and Susan F. Smith Center for Outcomes Research in Cardiology, Beth Israel Deaconess Medical Center Harvard Medical School Boston MA USA; ^9^ Harvard‐Thorndike Electrophysiology Institute, Beth Israel Deaconess Medical Center Harvard Medical School Boston MA USA; ^10^ Department of Cardiology Chelsea and Westminster Hospital NHS Foundation Trust London UK

**Keywords:** bundle‐branch block, clustering, ECG, machine learning, phenotyping, Electrophysiology

## Abstract

**Background:**

Abnormal ventricular depolarization, evident as a broad QRS complex on an ECG, is traditionally categorized into left bundle‐branch block (LBBB) and right bundle‐branch block or nonspecific intraventricular conduction delay. This categorization, although physiologically accurate, may fail to capture the nuances of diseases subtypes.

**Methods:**

We used unsupervised machine learning to identify and characterize novel broad QRS phenogroups. First, we trained a variational autoencoder on 1.1 million ECGs and discovered 51 latent features that showed high disentanglement and ECG reconstruction accuracy. We then extracted these features from 42 538 ECGs with QRS durations >120 milliseconds and employed a reversed graph embedding method to model population heterogeneity as a tree structure with different branches representing phenogroups.

**Results:**

Six phenogroups were identified, including phenogroups of right bundle‐branch block and LBBB with varying risk of cardiovascular disease and mortality. The higher risk right bundle‐branch block phenogroup exhibited increased risk of cardiovascular death (adjusted hazard ratio [aHR], 1.46 [1.30–1.63], *P*<0.0001) and all‐cause mortality (aHR, 1.24 [1.16–1.33], *P*<0.0001) compared with the baseline phenogroup. Within LBBB ECGs, tree position predicted future cardiovascular disease risk differentially. Additionally, for subjects with LBBB undergoing cardiac resynchronization therapy, tree position predicted cardiac resynchronization therapy response independent of covariates, including QRS duration (adjusted odds ratio [aOR], 0.47 [0.25–0.86], *P*<0.05).

**Conclusions:**

Our findings challenge the current paradigm, highlighting the potential for these phenogroups to enhance cardiac resynchronization therapy patient selection for subjects with LBBB and guide investigation and follow‐up strategies for subjects with higher risk right bundle‐branch block.

## Graphical abstract

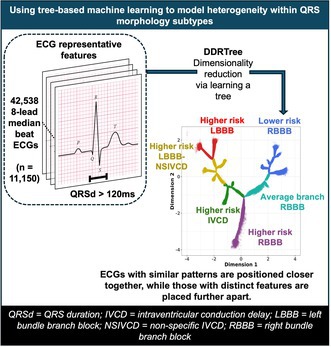

**Nonstandard Abbreviations and Acronyms**
BIDMC: Beth Israel Deaconess Medical Center
CRT: cardiac resynchronization therapy
DDRTree: dimensionality reduction via learning a tree
LAFB: left anterior fascicular block
LPFB: left posterior fascicular block
LVESD: left ventricular end‐systolic diameter
LVESV: left ventricular end‐systolic volume
LVSD: left ventricular systolic diameter
NSIVCD: nonspecific intraventricular conduction delay
UKB: UK Biobank
VAE: variational autoencoder
UVA: University of Virginia

**Clinical Perspective**

**What Is New?**

- We applied a tree‐based unsupervised machine learning approach to 42 538 broad QRS ECGs and identified 6 phenogroups within the conventional QRS morphology subtypes, each with distinct disease risk profiles, including a high‐risk right bundle‐branch block phenogroup.
- Using the tree dimensions, we quantified trends in future disease risk and cardiac resynchronization therapy response prediction and validated the approach on an external data set.

**What Are the Clinical Implications?**

- We demonstrate that using artificial intelligence‐enhanced electrocardiography in this unsupervised exploratory approach provides a granular understanding of QRS morphology subtypes, supporting personalized approaches to manage high‐risk patients and improving cardiac resynchronization therapy patient selection by identifying traits associated with cardiac resynchronization therapy responders.

Ventricular depolarization is initiated by specialized conduction tissue, the His‐Purkinje system, leading to rapid, synchronous contraction. In the ECG, this manifests as the QRS complex, which in healthy individuals is narrow and <120 ms in duration.

In certain pathologies or due to age‐related changes, there is abnormal ventricular depolarization, caused either by disease in the His‐Purkinje system or slowed myocardial cell‐to‐cell conduction. This manifests as prolonged QRS duration on the ECG. The ECG classification of abnormal ventricular depolarization is somewhat simplistic, grounded in century‐old conventions.^1^ These conventions use an arbitrary threshold of 120 ms and broadly categorize the abnormalities into left bundle‐branch block (LBBB), right BBB (RBBB), and nonspecific intraventricular conduction delay (NSIVCD) based on gross QRS morphology. Despite their limitations, these classifications have been used for risk stratification and to select patients for treatments such as cardiac resynchronization therapy (CRT) and implantable cardioverter‐defibrillators.^2^, ^3^, ^4^, ^5^, ^6^ Unsurprisingly, these criteria are imperfect, which may contribute to CRT non‐response.^7^

Advances in artificial intelligence (AI) now allow for new approaches to ECG analysis, detecting subtle abnormalities and performing diagnostic and predictive tasks^8^, ^9^, ^10^, ^11^ with high accuracy. In this study, we employed AI‐ECG analyses in an unsupervised manner to derive various phenogroups of broad QRS complexes, thereby redefining abnormal ventricular depolarization. This was achieved by deriving representational ECG features using a variational autoencoder (VAE) and applying dimensionality reduction via learning a tree (DDRTree)^12^ to create a compact, tree‐structured 2‐dimensional representation of the underlying population variation. The tree captures mixed phenotypes at its center, with more distinct phenotypes located toward the distal ends of the branches. This enables precise phenogrouping of broad QRS conduction disease, which may guide investigations and therapeutic interventions.

## METHODS

UK Biobank (UKB) data are available upon application (http://www.ukbiobank.ac.uk/). The Beth Israel Deaconess Medical Center (BIDMC) and University of Virginia (UVA) data sets are restricted due to ethical limitations. The programming code relating to creating the DDRTree structure and projecting other data onto the tree is available at this (GitHub repository).

### Cohorts

#### Beth Israel Deaconess Medical Center

BIDMC is a secondary care data set comprising routinely collected data from Boston, MA, USA. Subjects >16 years old with valid ECGs collected between 2000 to 2023 were included. There were 1 163 401 available ECGs collected from secondary care patients attending BIDMC. ECGs were linked to mortality records and other disease outcomes based on *International Classification of Diseases, Ninth Revision* (*ICD‐9*) and *Tenth Revision* (*ICD‐10*) codes. A subset of BIDMC patients underwent CRT, providing data on echocardiography measures taken before and 6 months after the procedure, which were used to define CRT response end points. The BIDMC cohort served as the main derivation cohort to create the broad QRS DDRTree trajectory in this work.

#### UK Biobank

The UKB^13^ cohort is a longitudinal study involving more than 500 000 volunteers aged 40 to 69 at enrolment between 2006 and 2010; this cohort represents a healthy volunteer population.^14^ It incorporates data from electronic health records, as well as interviews and questionnaires conducted at baseline. A subset of UKB participants from the original cohort underwent further investigation with digital ECGs with available ECG measurements (n=65 162). Outcomes were linked to death registry data, cancer records, hospital admissions, and primary care records. This cohort served as the external validation population for the DDRTree approach implemented in the BIDMC cohort.

#### University of Virginia

A CRT study^15^ was conducted in 200 patients at UVA, Charlottesville, VA, USA. Patients were aged between 40 and 89 years old, had a class I or II guideline for CRT, and had ECGs available before CRT. Before receiving CRT, patients provided information on demographics, comorbidities and medications and underwent echocardiography and ECG recordings. At 6 months post‐CRT, repeat instances of echocardiography were taken to define response end points. This cohort was used for the external validation of the BIDMC CRT analysis.

### Ethical Approvals

This study complies with all relevant ethical regulations. For the BIDMC, cohort ethics review and approval were provided by the BIDMC committee on Clinical Investigations, institutional review board protocol #2023P000042. The UKB received approval from the North West Multi‐Centre Research Ethics Committee as a Research Tissue Bank (application ID 48666). Patients provided informed consent that was approved by the University of Virginia Institutional Review Board for Human Subjects Research.

### ECG Preprocessing

The 12‐lead ECGs were pre‐processed using a bandpass filter 0.5 to 100 Hz, notch filter at 60 Hz, and resampling to 400 Hz. Single median beats were created from 10s ECGs using only 8 leads (excluding aVL, aVR, aVF, and lead III, because these are linear combinations of leads I and II) using the BRAVEHEART ECG analysis software.^16^

### VAE Model

A VAE was trained based on a convolutional encoder/decoder architecture previously described and used for ECG analysis.^17^, ^18^ In particular, the encoder composed of 6 convolutional blocks of feature extraction, adjusted for the median beat ECG signal, with the decoder designed as a symmetrically inverse network. The total number of parameters were 1 533 888 and 1 283 976 for the encoder and decoder, respectively. Further detail regarding model training and hyperparameter specifications are described in Data S1. The VAE was trained using only median beats as input to extract 51 representative features that capture underlying ECG patterns within the population. These features are not predefined variables but mathematically derived representations of ECG signals, capturing axes of maximal variation. Because no outcome data were used to derive these features, the method adhered to an unsupervised approach.

The VAE model was trained on all available median beat ECGs for the BIDMC cohort.

For the UKB cohort, BIDMC CRT subset and UVA CRT data, the BIDMC VAE model was used only to extract representative features specific to these subsets without any further training or fine‐tuning. Hence, from each of the 3 cohorts, 51 representative features were available. Upon filtering these cohorts using the selection criteria (detailed later), 51 representative features were selected for the filtered broad QRS subsets.

### Dimensionality Reduction via Learning a Tree

DDRTree is a dimensionality reduction method with reversed graph embedding^19^ that projects input data as a low‐dimensional tree structure with assigned branches representing clusters. This approach, originally described for single cell trajectories, has recently been successfully applied to clinical data for taxonomy of hypertrophic cardiomyopathy^20^ and type 2 diabetes.^21^ It places ECGs across the constructed tree such that those in close proximity share similar appearance whereas those further apart are more differentiated with distinct morphological features. We used the DDRTree implementation in the 2.22.0 version of monocle^22^ from the Bioconductor repository for all the analyses. The 51 latent features derived from the VAE for the broad QRS BIDMC population were entered into DDRTree for unsupervised clustering.

### Tree Construction

#### Selection Criteria

**Figure 1:**
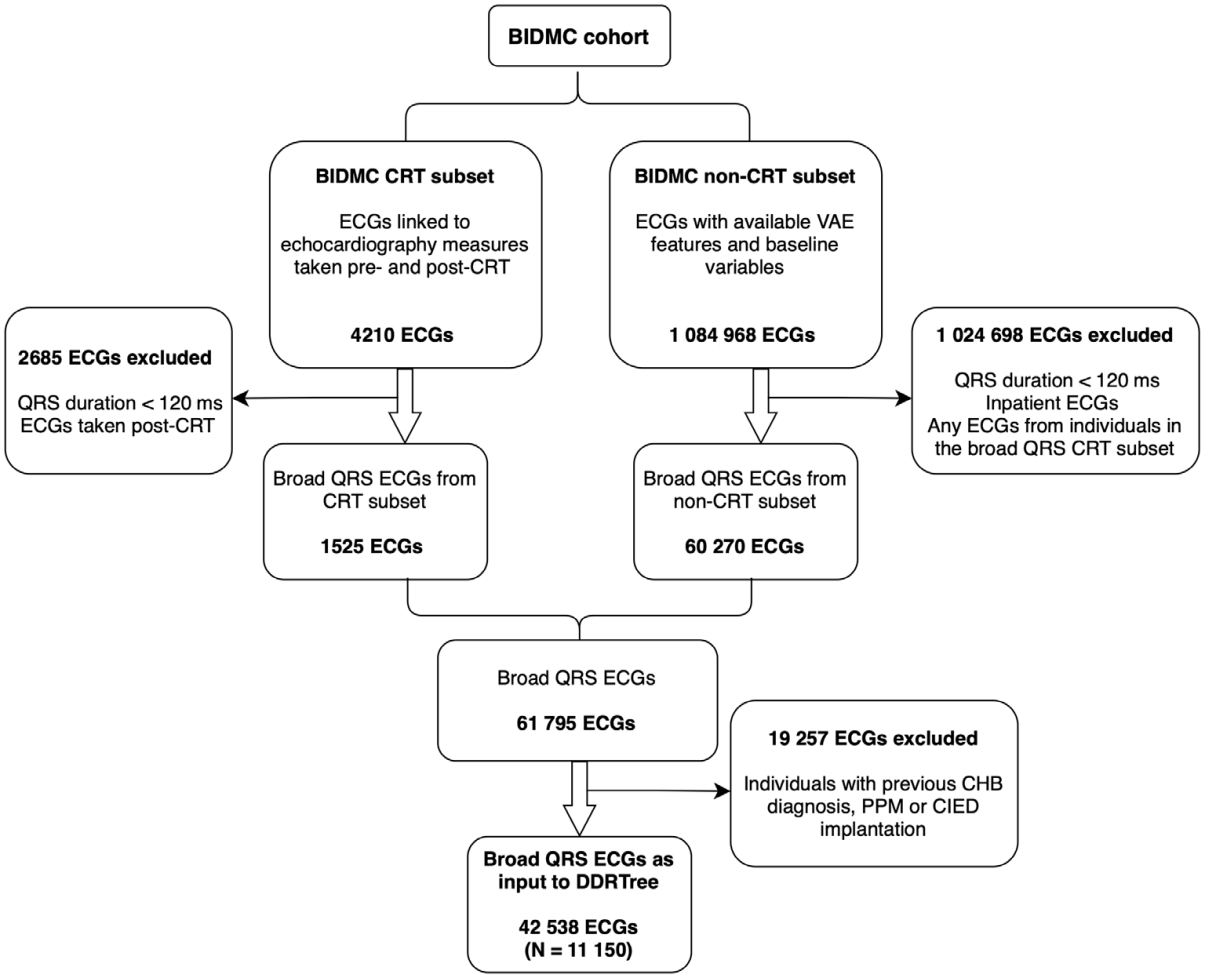
Flow chart of selection criteria for deriving the BIDMC broad QRS population for DDRTree analysis. BIDMC indicates Beth Israel Deaconess Medical Center; CHB, complete heart block; CIED, cardiovascular implantable electronic device; CRT, cardiac resynchronization therapy; DDRTree, dimensionality reduction via learning a tree; PPM, permanent pacemaker; and VAE, variational autoencoder.

We derived the main broad QRS DDRTree from the BIDMC cohort. The selection criteria applied to the BIDMC CRT subset and non‐CRT subset, used to derive the BIDMC broad QRS data set, are visualized as a flow chart in Figure 1. In total, 42 538 ECGs from 11 150 unique individuals were available to use across both subsets including a final of 1296 ECGs in the BIDMC CRT subset. Selection criteria for the UKB cohort was simpler given its healthy volunteer characteristics, filtering for QRS durations >120 ms resulting in 2319 available ECGs. For the UVA cohort, selection criteria included digital ECGs, nonright ventricle paced with LBBB morphology, resulting in 141 available ECGs for the CRT analysis. Demographics for the resulting subsets across the different cohorts are presented in Tables S1 and S2.

#### Retrieving the Tree Trajectory Using DDRTree

The main DDRTree trajectory was constructed using the VAE features from the broad QRS BIDMC cohort. As required by the DDRTree algorithm, these features were normalized before being input into DDRTree with standard parameters. Different centroid values were explored to balance sufficient complexity of the tree and stable branch allocation with computational complexity of the k‐means algorithm. As a result 2000 centroids per iteration were selected. The model returned DDRTree branch assignments, coordinates for dimensions 1 and 2 and pseudotime, representing the distance from the center of the tree. The internal validation of the constructed tree showed a median adjusted Rand Index of 0.57 (0.47–0.67).

To characterize the branches meaningfully and with sufficient statistical power, these were merged to create larger, representative branches/phenogroups based on spatial proximity of subbranches as described in Data S1. IVCD labels (RBBB, LBBB, NSIVCD) were predicted using the deep neural network^23^ model for the BIDMC non‐CRT subset because QRS morphology labels were available for the CRT data. The NSIVCD label was derived from the absence of LBBB and RBBB labels.

#### External Validation

The broad QRS UKB and UVA CRT cohorts served as independent external validation populations in this study. These cohorts were completely excluded from the development of the BIDMC tree and were independently mapped onto the derived BIDMC DDRTree without any further fine‐tuning of the trajectory or reclustering using DDRTree. Tree dimension coordinates and phenogroup assignments were predicted using supervised machine learning models trained on variables from the BIDMC DDRTree. A 2‐step approach was chosen to predict the coordinates: (1) independent Extreme Gradient Boosting (XGBoost) regression models to predict coordinates for DDRTree dimensions, and (2) a distance‐estimating algorithm, previously described by Nair et al.^21^ XGBoost models were trained on the latent ECG features from the BIDMC cohort. Using a train/validation split of 75:25, performance metrics on the validation BIDMC split were *R* ^2^_dim 1_=0.96, mean absolute error_dim 1_=0.29, *R* ^2^_dim 2_=0.91 and mean absolute error_dim 2_=0.31. Phenogroup assignments were predicted using a K Nearest Neighbor model, with tree coordinates serving as input. Initially, 10‐fold cross‐validation determined the optimal number of K neighbors (exploring a range of 1–200 neighbors) based on the best performance on the internal holdout validation set, using precision, recall, and F1‐score as scoring metrics. K neighbors of 3 yielded the best overall performance (Figure S1) and were then employed to train models for predicting phenogroup assignments for the UKB and UVA cohorts. The QRS morphology labels were also predicted for the UKB cohort; as mentioned previously, these were already available for the UVA cohort.

### Outcome Definition

#### CRT End Points

CRT response likelihood was evaluated based on 3 end points: a 10% increase in left ventricular ejection fraction (LVEF), a 15% decrease in LV end‐systolic diameter (LVESD), or a 15% decrease in LV end‐systolic volume (LVESV). These end points represent the absolute difference for LVEF and the relative differences for LVESD and LVESV between pre‐CRT and 6‐month post‐CRT measurements.

#### Diagnostic and Imaging Data

*ICD‐9* and *ICD‐10* codes were used to defined presence of disease in the BIDMC and UKB cohorts. For the diagnosis of atrial fibrillation (AF), either *ICD* code or an ECG showing AF was used. Echocardiograms were available for a subset of BIDMC participants; those within 60 days of an ECG were linked and used to assess associations of tree variables to structural changes.

### Statistical Analysis

For descriptive analyses, continuous values were represented as median values with interquartile ranges and categorical data were expressed as frequencies and percentages. Differences in phenogroup characteristics were assessed using Kruskal–Wallis rank sum test for continuous variables and Pearson's chi‐square tests for categorical variables. Alongside overlaying baseline and ECG variables across the tree, we further quantified these using univariate regressions across the phenogroups. We also assessed the spatial autocorrelation strength of these variables across the main BIDMC DDRTree trajectory using Global Moran's I. Multivariate logistic/linear regression models (depending on whether the outcome was binary or continuous) were used to assess associations between tree variables (phenogroup assignments and tree dimensions) and each of the following: echocardiography measures, CRT response end points, and prevalent disease outcomes. To evaluate heterogeneity in CRT response within a specific phenogroup for the BIDMC CRT subset, we also derived pseudotime as a measure of distance from the start of the branch moving toward its periphery. To assess the prognostic significance of the tree variables, time‐to‐event Cox proportional hazards models were used for both fatal end points, and Fine–Gray subdistribution hazard models were applied to the nonfatal outcomes, where instances of prevalent disease were filtered out and the competing risk of death was accounted for. The covariates adjusted for across these models comprised age, sex, ECG measures (heart rate, QTc interval and QRS duration), and QRS morphology (RBBB, LBBB, NSIVCD); for the CRT analysis the covariates adjusted for included age, sex, and QRS duration. We also conducted a sensitivity analysis by omitting QRS morphology as a covariate in both prevalent and incident disease models to assess the robustness of the associations between phenogroups and disease outcomes. Additionally, we compared the prognostic performance of the QRS morphology categories against the broad QRS phenogroups in the BIDMC cohort for all‐cause mortality, adjusting for age, sex, and ECG measures. We quantified the bootstrapped C‐indices from both models and assessed the goodness of fit across the 2 models.

Due to high missingness of ECG measures in the UKB cohort data, these were not adjusted for in the analyses conducted in the UKB cohort. We aimed to analyze overlapping cardiovascular and mortality outcomes across the BIDMC and UKB cohorts but were constrained by the availability of outcomes across the data sets. To account for the multiplicity of disease outcomes assessed in the BIDMC cohort, we applied multiple testing corrections to the main analyses as part of the sensitivity analyses. In line with recent findings suggesting the adequacy of well‐powered clinical data sets without the need for proportional hazards testing,^24^ we refrained from evaluating this assumption. Instead, we regarded the Cox model's hazard ratio (HR) as an average estimate across the follow‐up duration. Statistical analyses and hierarchical clustering models were built with R (version 4.2.0) and the VAE and supervised machine learning models were built with Python (version 3.9).

### Modeling Individual Disease Risk Using DDRTree‐Derived Variables

To obtain the individual probability of developing each cardiovascular and mortality outcome for each participant at median follow‐up time, we used Cox proportional hazard models for both fatal end points, and Fine‐Grey subdistribution hazard models for non‐fatal outcomes, where the competing risk of death was accounted for. All models were constructed using tree dimensions and pseudotime as input. As a result, we predicted individual‐level probability for the incidence of various diseases. These were overlaid across the tree structure to visualize the heterogeneity in cardiovascular disease and mortality risk progression.

### Explainability

For explainability, we plotted the average median beats per phenogroups across 12 leads to assess visual changes distinct to phenogroups. To assess how the latent features used to create DDRTree relate to the phenogroups, we employed multivariate logistic regression models with Elastic Net regularization regressing all 51 representative features against each phenogroup assignment. This regularization approach was chosen to deal with multicollinearity across the features in a flexible manner to allow shrinking nonpredictive coefficients to zero using least absolute shrinkage and selection operator penalization but also reduce the impact of correlated features through Ridge penalization. The approach of tuning the alpha parameter for Elastic Net and lambda value are described in Data S1. This approach allowed us to identify the top 3 most significant features while accounting for the influence of other factors and their associations with the phenogroups. Additionally, these features were visualized through latent transversals to provide further insight.^25^

## RESULTS

**Figure 2:**
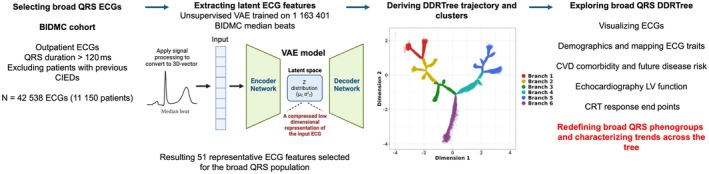
Summary of the main steps to model the broad QRS DDRTree trajectory on the BIDMC population and analyze trends across derived phenogroups and tree structure. BIDMC indicates Beth Israel Deaconess Medical Center; CIED, cardiovascular implantable electronic device; CRT, cardiac resynchronization therapy; CVD, cardiovascular disease; DDRTree, dimensionality reduction via learning a tree; LV, left ventricular; and VAE, variational autoencoder.

An overview of the main steps to model the broad QRS ECGs from the BIDMC population using DDRTree is summarized in Figure 2. To input interpretable ECG features into DDRTree, median beats from the full BIDMC cohort were first used to train a VAE model. This dimensionality reduction technique captured 51 axes of maximal variation (latent features) that can reconstruct ECGs with a median Pearson's r of 0.99. Derived latent features were then filtered for the broad QRS population as input to DDRTree; VAE reconstruction performance in this subpopulation also performed similarly (Figures S2 and S3), therefore representing the constituent parts of broad complex median beat ECGs. The resulting tree structure and the 6 representative branches identified across 2 reduced dimensions from DDRTree, with each phenogroup capturing distinct regions within the tree, are shown in Figure 2.

To externally validate the DDRTree approach, the broad QRS UKB subset was projected onto the tree derived from the BIDMC cohort by predicting the tree coordinates and phenogroup assignments in a supervised manner. The resulting visual arrangement of UKB ECGs closely followed the structure of the reference tree, with no significant difference in the distribution of correlations between the 2 cohorts, suggesting consistency in their projections on the tree (Figure S4).

### Visualizing Reconstructed ECGs and Associated Latent Features Across BIDMC Phenogroups

**Figure 3:**
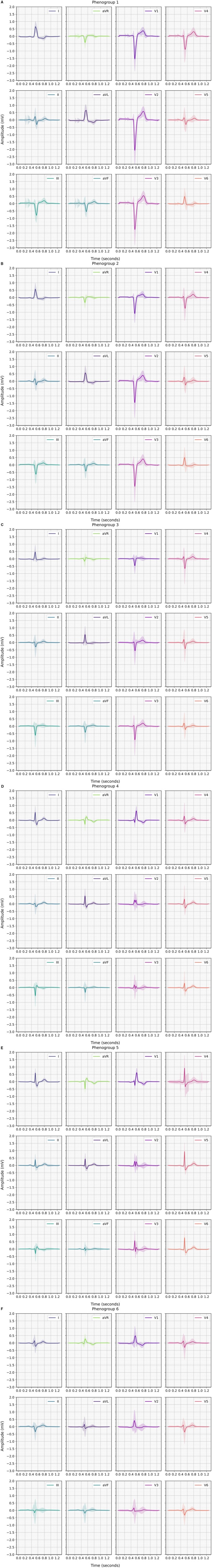
Visualizing the representative median beats from the BIDMC cohort across 12‐leads in the 6 broad QRS DDRTree phenogroups. **A–F** show the averaged 12‐lead median beat ECG constructed using across constituent ECGs within each phenogroup ranging from 1 to 6. BIDMC Beth Israel Deaconess Medical Center; and DDRTree, dimensionality reduction via learning a tree.

We first visualized the averaged median beats for the 12 leads for all ECGs in each phenogroup to display the representative signal per phenogroup and to assess morphological differences (Figure 3). Phenogroups 1 and 2 showed similar patterns of rS with deep S‐waves in leads V1 and V2 with ST elevation suggesting some LBBB morphology. These ECG traits were absent in the representative median beat for phenogroup 3. Phenogroups 4 and 5 also shared traits indicative of RBBB morphology with rsR' and rSR' appearance in leads V1 and V2 respectively and broad slurred S‐waves in V6. Lead V1 in the median beat signal from phenogroup 6 showed similar patterns of T‐wave inversion without the distinct R and S wave ECG findings seen in phenogroups 4 and 5. To interpret the phenogroups in the context of latent features, we conducted regression analyses of the latent factors against the phenogroups. The top 3 features per phenogroup and their associations are shown in Figure S5B, with latent traversals of these features in Figure S5C.

### Investigating Phenotypic Heterogeneity Across the Tree

#### Demographics Variables

Once we derived the tree of all broad QRS ECGs, we proceeded to explore the differences in phenogroups derived in this unsupervised manner and observed clear differences between the phenogroups. Table 1 shows the most important differences between the phenogroups in the BIDMC cohort across baseline variables, ECG measures and findings and echocardiography measures (detailed version in Table S3).

**Table 1:** Descriptive Statistics of the BIDMC Broad QRS DDRTree Phenogroups Across Baseline Variables, ECG, and Echocardiography Measures

Variable	Phenogroup 1, N=5902*	Phenogroup 2, N=8917*	Phenogroup 3, N=6145*	Phenogroup 4, N=4863*	Phenogroup 5, N=12123*	Phenogroup 6, N=4588*	*P* value†
Baseline variables							
Age, y	73 (65–82)	74 (65–81)	72 (63–79)	73 (65–81)	73 (65–80)	74 (66–82)	<0.001
Male sex	2859 (48%)	5421 (61%)	4689 (76%)	3440 (71%)	8568 (71%)	3402 (74%)	<0.001
Systolic blood pressure, mm Hg	132 (121–143)	130 (120–141)	128 (119–139)	132 (123–142)	133 (124–143)	129 (120–139)	<0.001
Diastolic blood pressure, mm Hg	70 (64–77)	71 (65–78)	72 (65–79)	72 (66–78)	72 (66–79)	71 (65–77)	<0.001
ECG measures							
Heart rate, bpm	66 (59–76)	69 (60–79)	68 (60–78)	68 (60–79)	65 (57–74)	70 (61–81)	<0.001
QRS interval, ms	148 (138–160)	138 (128–150)	130 (124–144)	140 (130–148)	140 (132–150)	144 (134–154)	<0.001
PR interval, ms	178 (160–202)	180 (160–206)	180 (156–208)	176 (154–200)	172 (152–194)	178 (158–203)	<0.001
QTc interval, ms	476 (456–496)	468 (446–490)	461 (438–485)	459 (439–481)	455 (436–477)	468 (446–490)	<0.001
QRS axis							
Normal QRS axis	1268 (38%)	2602 (44%)	1716 (39%)	1318 (37%)	4698 (60%)	523 (15%)	<0.001
Right axis deviation	14 (0.4%)	78 (1.3%)	172 (3.9%)	276 (7.8%)	692 (8.9%)	488 (14%)	<0.001
Left axis deviation	2010 (61%)	3095 (53%)	2251 (51%)	1689 (48%)	2200 (28%)	1896 (55%)	<0.001
Extreme axis deviation	23 (0.7%)	87 (1.5%)	275 (6.2%)	256 (7.2%)	220 (2.8%)	569 (16%)	<0.001
QRS morphology							<0.001
Nonspecific intraventricular conduction delay	751 (13%)	3072 (34%)	3990 (65%)	340 (7.0%)	598 (4.9%)	441 (9.6%)	
Left BBB	5145 (87%)	5740 (64%)	913 (15%)	6 (0.1%)	29 (0.2%)	58 (1.3%)	
Right BBB	4 (<0.1%)	102 (1.1%)	1237 (20%)	4515 (93%)	11 496 (95%)	4089 (89%)	
Echocardiography measures							
LV ejection fraction, %	46 (32–59)	48 (32–60)	53 (35–60)	60 (52–68)	60 (55–70)	57 (43–65)	<0.001
LV end‐diastolic diameter, mm	49 (43–56)	50 (44–57)	52 (46–58)	47 (42–52)	47 (42–52)	48 (42–54)	<0.001
LV end‐systolic diameter, mm	35 (29–45)	36 (29–46)	36 (30–44)	30 (25–35)	30 (26–34)	31 (25–37)	<0.001

Continuous variables are shown as median values with the associated interquartile ranges (25th–75th percentile) and categorical variables are shown with their frequencies. Differences across phenogroups were quantified using the nonparametric Kruskal–Wallis rank sum test and Pearson's chi‐square test for continuous and categorical variables, respectively using a p‐value significance threshold of 0.05. BBB indicates bundle‐branch block; BIDMC, Beth Medical Israel Deaconess Center; DDRTree, dimensionality reduction via learning a tree; and LV, left ventricular.

* Median (25%–75%); n (%).

^†^ Kruskal–Wallis rank sum test; Pearson's chi‐square test.

Phenogroup 1 had the highest median QRS duration and QTc interval, with 87% of constituent ECGs showing LBBB morphology (Table 1). Phenogroup 2 captured a mix of LBBB and NSIVCD labels and highest median PR interval. Phenogroup 3 captured majority of men, with 65% ECGs with NSIVCD labels and a mix of both BBB morphologies suggesting an IVCD phenotype. Phenogroups 4, 5, and 6 all had majority of RBBB morphology. DDRTree is known to construct the tree and order phenogroups based on the information captured such that mixed phenotypes are within the tree center while moving the distal ends of branches captures more distinct, extreme phenotypes. Given phenogroup 4 showed average traits comparative to other phenogroups and was located in the tree core (refer to Figure 2), it was defined as the average RBBB phenogroup and used as the reference group for downstream regression analyses. Phenogroup 5 consisted of a majority of ECGs with normal QRS axis and shared the highest median LVEF of 60% with phenogroup 4. A majority of ECGs within phenogroup 6 had abnormal QRS axis with the highest frequency of right, left, and extreme axis deviation. Similar profiles were observed within the UKB population (Table S4) across the projected phenogroups, with the majority of ECGs falling within phenogroup 5, as seen in the BIDMC cohort. Through initial descriptive analyses, it became evident that a spectrum of phenotypes exists within the recognized classification of QRS morphology.

#### Overlaying ECG Measures Across the Derived DDRTree Trajectory

**Figure 4:**
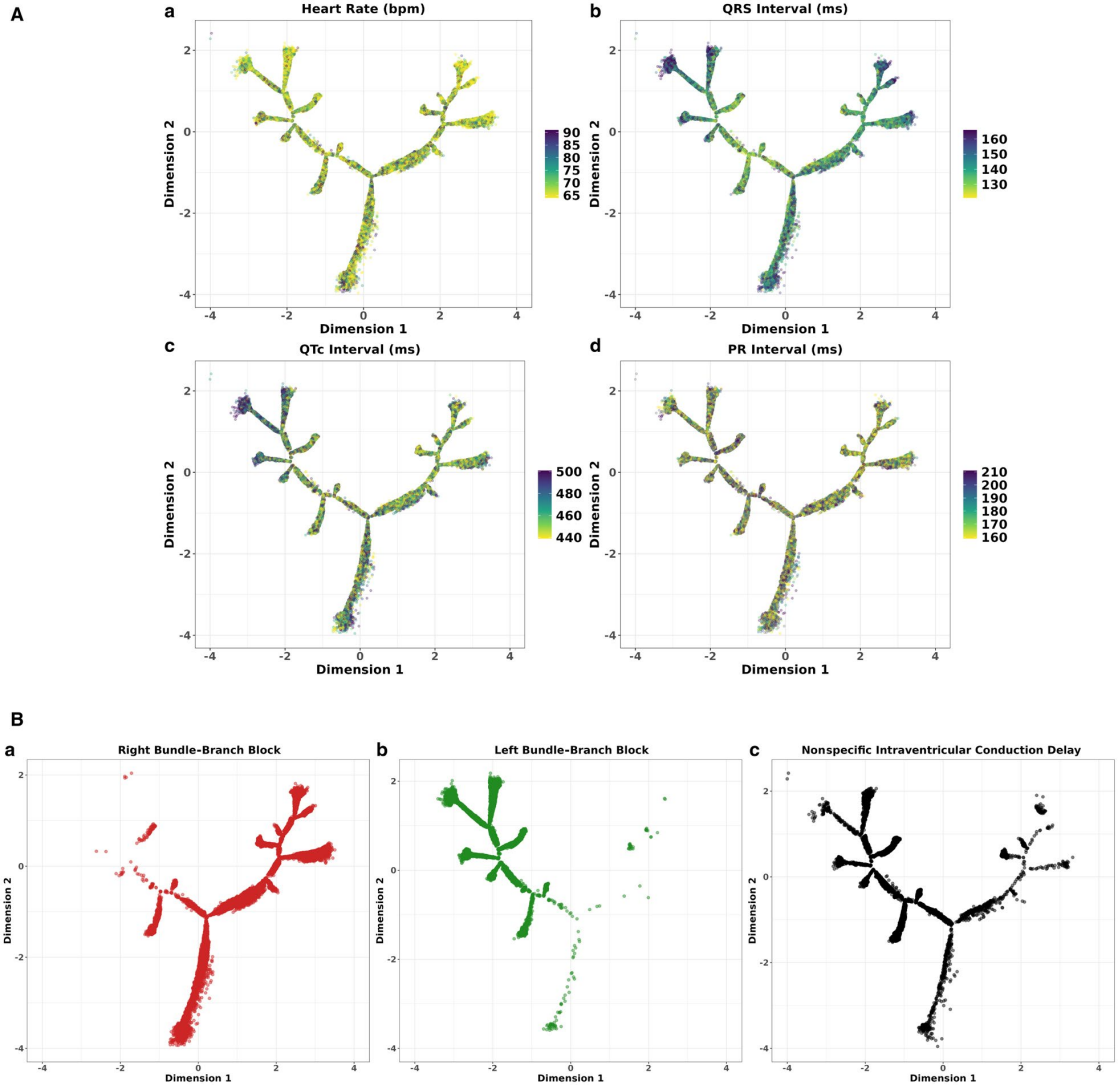
Overlaying ECG measures and QRS morphology labels across the broad QRS BIDMC DDRTree. **A**, Heart rate, QRS, QTc, and PR intervals were overlaid across the tree in subfigures (**a** **–d**). To enhance the clarity of the visualizations, the scales of the values plotted were limited to emphasize values within a specific range, indicated by the respective legends. **B,** Different forms of IVCD such as right and left bundle‐branch block and nonspecific IVCD were overlaid across the tree in (**a–c**). BIDMC Beth Israel Deaconess Medical Center; DDRTree, dimensionality reduction via learning a tree; and IVCD, intraventricular conduction delay.

Using the tree dimensions, we mapped ECG variables from the ECGs across the tree in Figure 4. These trends were additionally quantified by univariate regressions of baseline variables against phenogroup assignments in Figure S6. We also quantified the spatial autocorrelation of these variables to evaluate whether their distribution was meaningfully clustered across the tree (Figure S7). Most variables showed strong autocorrelation (*P*<0.01 across all) with the highest Global Moran's I estimate was observed for RBBB followed by LBBB, NSIVCD labels, and QRS duration. Higher values of QRS and QTc intervals were seen in the top‐left region (low dimension 1, high dimension 2), increasing toward the branch periphery and decreasing toward the tree core (Figure 4A). LBBB ECGs were also predominant within this region (Figure 4B). The greatest density of RBBB ECGs appeared in regions with few LBBB ECGs, making the transition from LBBB to RBBB clear while NSIVCD ECGs were distributed across the tree.

#### Comorbidity Burden and Future Risk of Disease

**Figure 5:**
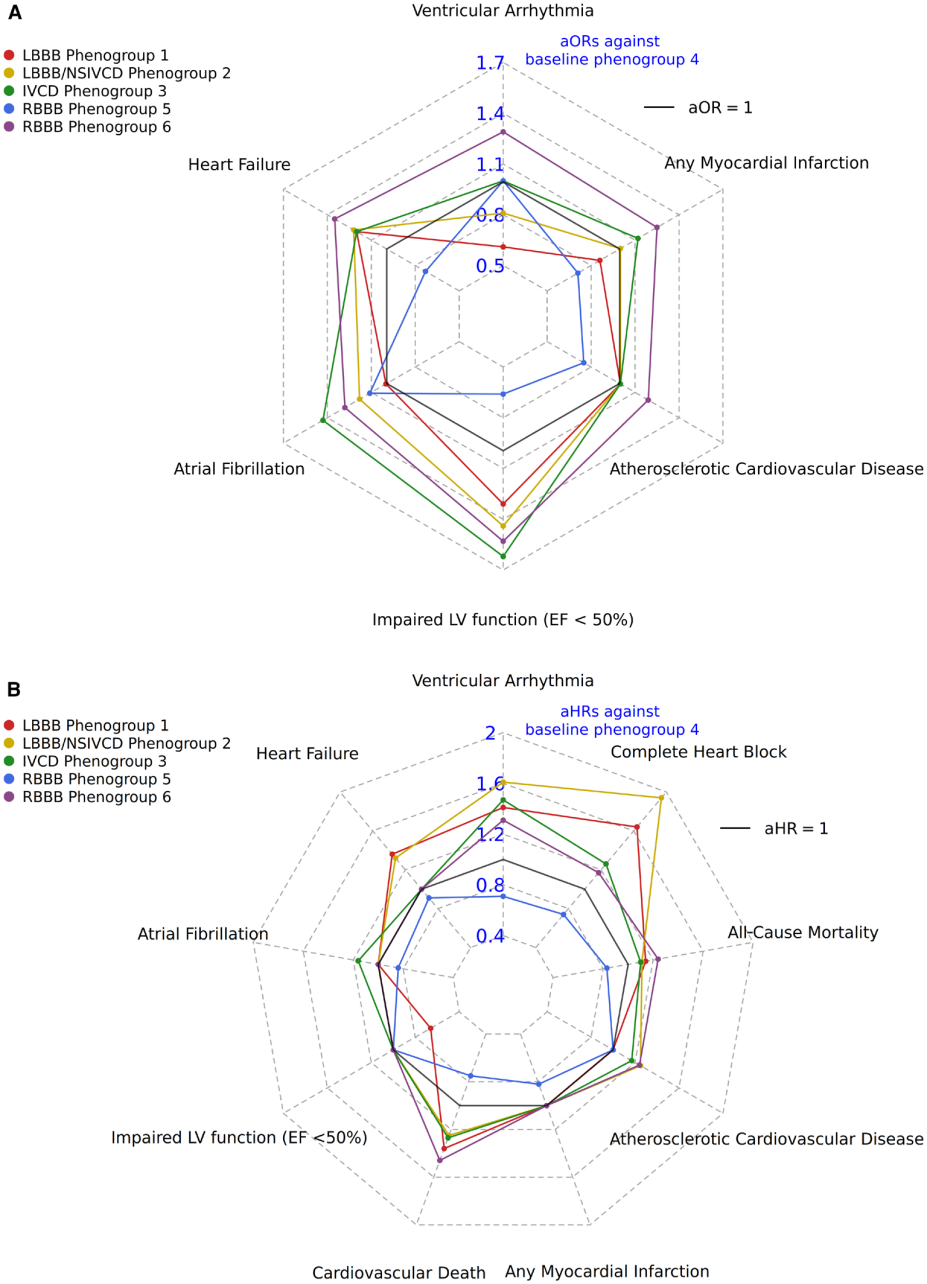
Investigating heterogeneity within prevalent comorbidities and future risk of cardiovascular diseases across the broad QRS BIDMC DDRTree. **A**, Radar plot showing the adjusted exponentiated odds ratio estimates for the different phenogroups against each prevalent disease outcome using phenogroup 4 as the baseline comparator. The regression estimates that did not reach statistical significance (*P* ‐value >0.05) were set at an aOR of 1 in the plot. **B**, Radar plot showing adjusted exponentiated hazards ratio estimates for the different phenogroups against each incident disease outcome using phenogroup 4 as the baseline comparator. Cox proportional hazards (HR) are reported for the fatal outcomes and Fine–Gray SHRs are reported for the nonfatal outcomes, to account for the competing risk of death. The regression estimates that did not reach statistical significance (*P* value >0.05) were set at an aHR of 1 in the plot. All regression models presented were adjusted for age, sex, heart rate, QRS, and QTc interval and type of QRS morphology (right or left bundle‐branch block, nonspecific intraventricular conduction delay). Death was treated as the competing event for other incident outcomes. aOR indicates adjusted exponentiated odds ratio; aHR indicates adjusted hazards ratio; BIDMC, Beth Medical Israel Deaconess Center; DDRTree, dimensionality reduction via learning a tree; EF, ≤50% impaired left ventricular function (ejection fraction <50%); IVCD, intraventricular conduction delay; LBBB, left bundle‐branch block; LV, left ventricular; NSIVCD, nonspecific intraventricular conduction delay; and RBBB, right bundle‐branch block.

We next aimed to characterize the QRS morphology phenogroups and tree trajectory further based on trends in cardiovascular comorbidity burden and incident disease risk. The event rates for the incident diseases in the BIDMC cohort can be found in Table S5; the median follow‐up time across the cohort was 1398 days. The resulting estimates for the phenogroups from the prevalent disease models and incident disease models can be found in Tables 2 and 3, respectively. For interpretability, only significant associations and the respective regression estimates (nonsignificant estimates indicated as odds ratio (OR)/HR of 1) have been plotted within radar plots in Figure 5. Radar plots with the regression estimates and the 95% CIs are plotted in Figure S8. The regression estimates of disease outcomes against tree dimensions are shown in Figure S9.

**Table 2:** Adjusted Odds Ratios From Multivariate Logistic Models Evaluating the Associations Between Phenogroup Assignments and Prevalent Cardiovascular Disease Outcomes in the BIDMC Broad QRS DDRTree Population

Phenogroup	Exponentiated odds ratio (95% CIs) *P* value					
	VA	HF	EF ≤50%	ASCVD	MI	AF
LBBB phenogroup 1	0.61(0.51–0.73), *P*<0.0001	1.20 (1.06–1.36), *P*<0.01	1.31 (1.14–1.50), *P*<0.001	1.06 (0.93–1.21), 0.35	0.86 (0.75–0.98), *P*<0.05	0.93 (0.82–1.05), 0.25
LBBB‐NSIVCD phenogroup 2	0.81 (0.69–0.95), *P*<0.05	1.22 (1.09–1.37), *P*<0.001	1.44 (1.27–1.64), *P*<0.0001	1.05 (0.94–1.18), 0.38	1.03 (0.91–1.16), 0.65	1.18 (1.06–1.32), *P*<0.01
IVCD phenogroup 3	1.11 (0.96–1.28), 0.17	1.20 (1.08–1.32), *P*<0.001	1.62 (1.45–1.82), *P*<0.0001	1.02 (0.92–1.13), 0.76	1.12 (1.01–1.25), *P*<0.05	1.43 (1.30–1.58), *P*<0.0001
RBBB phenogroup 5	0.90 (0.80–1.02), 0.09	0.73 (0.68–0.79), *P*<0.0001	0.66 (0.61–0.73), *P*<0.0001	0.75 (0.69–0.80), *P*<0.0001	0.71 (0.66–0.77), *P*<0.0001	1.11 (1.04–1.19), *P*<0.01
RBBB phenogroup 6	1.29 (1.13–1.47), *P*<0.001	1.35 (1.24–1.47), *P*<0.0001	1.53 (1.39–1.69), *P*<0.0001	1.19 (1.09–1.31), *P*<0.001	1.25 (1.15–1.37), *P*<0.0001	1.28 (1.17–1.39), *P*<0.0001

Exponentiated odds ratios, 95% CIs, and associated *P* values are presented for each phenogroup, against the baseline comparator RBBB phenogroup 4, adjusted for covariates. Covariates included age, sex, ECG measurements (heart rate, QRS duration, QTc interval), and type of QRS morphology (LBBB/RBBB or NSIVCD). Statistical significance was set at < 0.05. AF indicates atrial fibrillation; ASCVD, atherosclerotic cardiovascular disease; BIDMC indicates Beth Israel Deaconess Medical Center; DDRTree, dimensionality reduction via learning a tree; EF, ≤50% impaired left ventricular function (ejection fraction <50%); HF, heart failure; IVCD, intraventricular conduction delay; LBBB, left bundle‐branch block; MI, myocardial infarction; NSIVCD, nonspecific IVCD; RBBB, right bundle‐branch block; and VA, ventricular arrythmia.

**Table 3:** Adjusted Hazard Ratios From Multivariate Time‐to‐Event Models Evaluating the Associations Between Phenogroup Assignments and Incident Cardiovascular Disease and Mortality Outcomes in the BIDMC Broad QRS DDRTree Population

Phenogroup	VA: SHR (95% CI, *P* value)	HF: SHR (95% CI, *P* value)	AF: SHR (95% CI, *P* value)	EF ≤50: SHR (95% CI, *P* value)	ASCVD: SHR (95% CI, *P* value)	MI: SHR (95% CI, *P* value)	Cardiovascular death: HR (95% CI, *P* value)	CHB: SHR (95% CI, *P*‐value)	Mortality: HR (95% CI, *P* value)
LBBB phenogroup 1	1.41 (1.13–1.76), *P*<0.01	1.36 (1.16–1.58), *P*<0.001	0.95 (0.80–1.13), 0.58	0.66 (0.44–0.98), *P*<0.05	1.14 (0.94–1.38), 0.18	1.17 (0.98–1.41), 0.09	1.36 (1.15–1.61), *P*<0.001	1.64 (1.33–2.03), *P*<0.0001	1.14 (1.03–1.27), *P*<0.05
LBBB‐NSIVCD phenogroup 2	1.61 (1.32–1.98), *P*<0.0001	1.32 (1.16–1.51), *P*<0.0001	0.96 (0.81–1.12), 0.58	1.08 (0.75–1.54), 0.69	1.25 (1.06–1.48), *P*<0.01	1.14 (0.96–1.35), 0.13	1.25 (1.07–1.47), *P*<0.01	1.94 (1.61–2.33), *P*<0.0001	1.11 (1.01–1.22), *P*<0.05
IVCD phenogroup 3	1.47 (1.23–1.76), *P*<0.0001	1.11 (0.99–1.25), 0.07	1.16 (1.01–1.33), *P*<0.05	1.10 (0.80–1.51), 0.55	1.17 (1.01–1.35), *P*<0.05	1.05 (0.91–1.22), 0.50	1.27 (1.11–1.45), *P*< 0.001	1.26 (1.07–1.49), *P*<0.01	1.10 (1.01–1.19), *P*<0.05
RBBB phenogroup 5	0.71 (0.61–0.82), *P*<0.0001	0.91 (0.84–0.99), *P*<0.05	0.84 (0.76–0.92), *P*<0.001	0.80 (0.63–1.01), 0.06	1.01 (0.91–1.12), 0.87	0.82 (0.74–0.92), *P*<0.001	0.75 (0.68–0.83), *P*<0.0001	0.74 (0.66–0.84), *P*<0.0001	0.83 (0.78–0.89), *P*<0.0001
RBBB phenogroup 6	1.31 (1.12–1.53), *P*<0.001	1.05 (0.95–1.16), 0.36	1.06 (0.95–1.19), 0.30	1.01 (0.76–1.35), 0.93	1.24 (1.09–1.42), *P*<0.01	0.98 (0.86–1.12), 0.79	1.46 (1.30–1.63), *P*<0.0001	1.17 (1.02–1.34), *P*<0.05	1.24 (1.16–1.33), *P*<0.0001

Cox proportional hazards (HR) are reported for the fatal outcomes and Fine–Gray SHR are reported for the nonfatal outcomes, to account for the competing risk of death. Exponentiated hazards ratios, 95% CIs, and associated *P* values are presented for each phenogroup, against baseline comparator RBBB phenogroup 4, adjusted for covariates. Covariates included age, sex, ECG measurements (heart rate, QRS duration, QTc interval) and type of QRS morphology (LBBB/RBBB or NSIVCD). All‐cause mortality was treated as the competing event for other incident outcomes. Statistical significance was set at *P* < 0.05. AF indicates atrial fibrillation; ASCVD, atherosclerotic cardiovascular disease; BIDMC, Beth Medical Israel Deaconess Center; CHB, complete heart block; DDRTree, dimensionality reduction via learning a tree; EF, ≤50% impaired left ventricular function (ejection fraction <50%); HF, heart failure; IVCD, intraventricular conduction delay; LBBB, left bundle‐branch block; MI, myocardial infarction; NSIVCD, nonspecific IVCD; RBBB, right bundle‐branch block; SHR, subdistribution hazards; and VA, ventricular arrythmia.

Both LBBB‐dominant phenogroups 1 and 2 were similarly associated with increased odds of prevalent LV systolic dysfunction (LVSD) and prevalent clinical heart failure (HF) compared with baseline (Table 2, Figure 5A). They similarly captured a higher risk of future disease across most outcomes but differed in their future risk of LVSD with LBBB phenogroup 1 being associated with 34% (adjusted subdistribution HR, 0.66 [95% CI, 0.44–0.98], *P*<0.05) lower risk of future LVSD whereas phenogroup 2 did not show a significant difference in risk compared with the baseline group (Table 3, Figure 5B). NSIVCD‐dominant phenogroup 3 was associated with increased odds of prevalent disease and elevated risk of future disease across the majority of outcomes with the highest risk for future AF indicating a high comorbidity burden and risk phenotype. RBBB‐dominant phenogroups 5 and 6 exhibited significant differences in comorbidity and future disease risk. Phenogroup 5 demonstrated a lower burden and risk profile across several outcomes compared with baseline, hence maintaining its low disease risk profile as described previously. Conversely, phenogroup 6 showed significantly increased odds of prevalent disease for several cardiovascular outcomes. Additionally, it was associated with elevated risk of future events, particularly for cardiovascular death (adjusted HR [aHR], 1.46 [95% CI, 1.30–1.63], *P*<0.0001) and all‐cause mortality (aHR, 1.24 [95% CI, 1.16–1.33], *P*<0.0001) (Table 3, Figure 5B). These trends were retrained in the sensitivity analyses correcting for multiple testing (Tables S6 and S7). In the sensitivity analysis assessing the impact of QRS morphology labels as a covariate (Tables S8 and S9), the overall trends across the phenogroups remained largely consistent. However, LBBB phenogroup 1 was associated with an increased future risk of LVSD. Regression estimates for the prevalent and incident analyses were higher for the LBBB phenogroups, whereas estimates for phenogroups 5 and 6 remained largely unchanged across both analyses. Trends seen against the tree dimensions aligned with phenogroup traits (Figure S9). The top‐right (positive dimension 1 and 2) region captured low comorbidity burden and low risk of future disease across majority of outcomes, aligning with the position of phenogroup 5.

We also assessed the prognostic power of broad QRS DDRTree phenogroups against QRS morphology categories for all‐cause mortality. Using 1000 bootstrapped iterations, the broad QRS phenogroup model achieved an average C‐index of 0.705 (SD=0.00239), and the QRS morphology model achieved 0.701 (SD=0.00252). The partial likelihood ratio test was used to compare the goodness of fit across the 2 models. The result suggested that the model fits were significantly distinguishable (*P*<0.0001), with the broad QRS DDRTree phenogroup model demonstrating better discrimination than the QRS morphology‐basedphenogroup model (*z*=6.69, *P*<0.0001).

The event rates for the incident diseases in the UKB cohort can be found in Table S10; the median follow‐up time across the cohort was 1049 days. Similar trends of disease burden and risk profiles across phenogroups and tree regions were observed in the UKB cohort (Tables S11 and S12, Figure S10). RBBB‐dominant phenogroup 5 within the UKB cohort showed significantly lower odds of prevalent myocardial infarction and a reduced risk of future AF (Tables S11 and S12). Conversely, LBBB‐dominant phenogroups 1 and 2 and the NSIVCD‐dominant phenogroup 3 maintained their association with increased prevalent HF burden.

**Figure 6:**
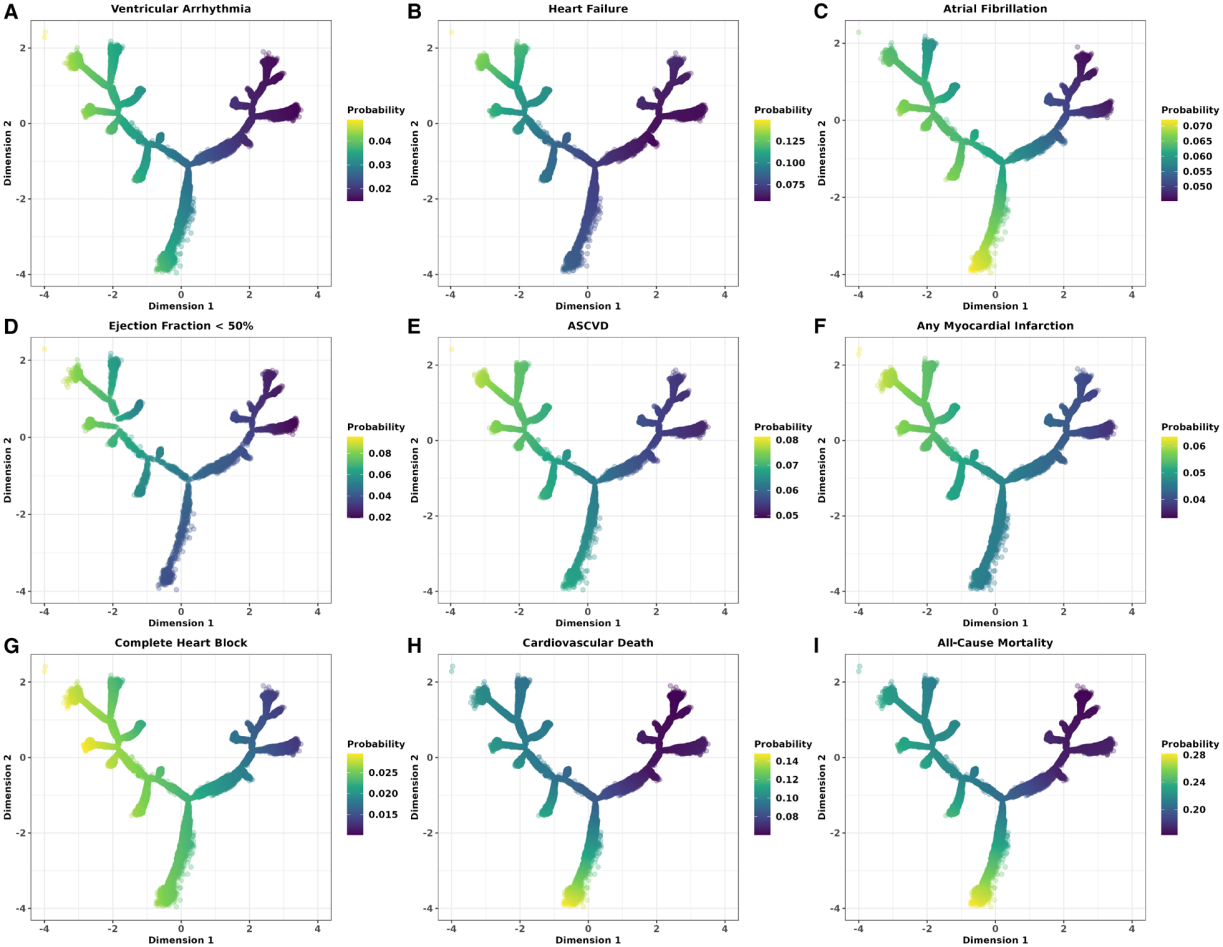
Overlaying predicted survival time plots for cardiovascular disease and mortality outcomes across the BIDMC broad QRS DDRTree. **A–I** show the overlaid risk probabilities for the different outcomes. These were estimated for individual samples within the tree, using Cox proportional hazards models for fatal outcomes and Fine–Gray subdistribution hazards models for nonfatal outcomes accounting for the competing risk of death, with tree dimensions and pseudotime (position within the tree, relative to the tree core) as inputs. All‐cause mortality was treated as the competing event for other incident outcomes. ASCVD indicates atherosclerotic cardiovascular disease; BIDMC, Beth Medical Israel Deaconess Center; and DDRTree, dimensionality reduction via learning a tree.

We also produced predicted survival time plots using Cox models (Figure 6) in the BIDMC population to demonstrate the utility of tree outputs in predicting individual risk probabilities for participants within the tree. These showed increasing probability of risk moving towards the left region of the tree across all outcomes. A distinctly greater risk of LVSD and HF was predicted in the top‐left region, where the predominantly LBBB ECGs were positioned, indicating disease risk predictions using tree‐derived variables align with trends seen within the general population as well. Additionally, the bottom region of the tree near the branch periphery uniquely captured a higher risk of mortality, aligning with the position of RBBB phenogroup 6.

### Redefining the Broad QRS Phenogroups

We were able to demonstrate that traditional classifications fail to capture the full spectrum of phenotypic variation within the broad QRS population. For instance, both LBBB and RBBB were found to consist of multiple subgroups with varying risk profiles.

**Figure 7:**
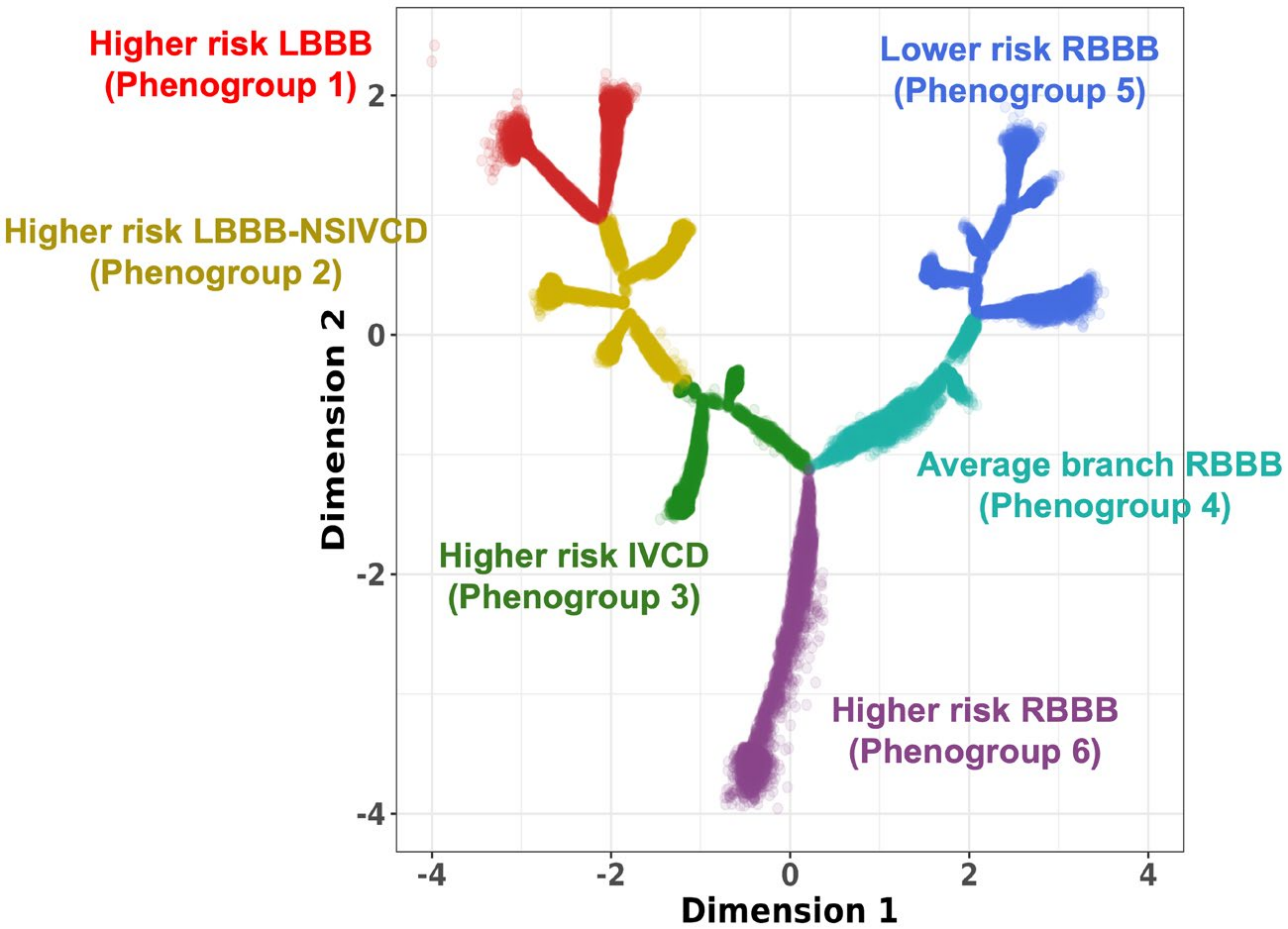
Six phenogroups identified within the broad QRS DDRTree population. Phenogroups were labeled by risk profile (cardiovascular disease, mortality) and most frequent QRS morphology type, each indicated by a different color. BIDMC indicates Beth Medical Israel Deaconess Center; DDRTree, dimensionality reduction via learning a tree; IVCD, intraventricular conduction delay, LBBB, left bundle‐branch block; NSIVCD, nonspecific intraventricular conduction delay; RBBB, right bundle‐branch block.

As a result, 6 distinct phenogroups were identified within this phenomapping approach. These were labeled by each phenogroup's risk profiles for cardiovascular diseases and mortality outcomes and their dominant QRS morphology (Figure 7). The following phenogroups were identified: (1) higher risk LBBB, (2) higher risk LBBB‐NSIVCD, (3) higher risk IVCD, (4) average branch RBBB, (5) lower risk RBBB, and (6) higher risk RBBB, as indicated by different colors. To visualize how ECGs vary across subregions of these phenogroups, we created an interactive tool to reconstruct median beat signals using VAE features within a selected region.

### Differential Disease Risk Patterns Across LBBB ECGs

LBBB‐dominant phenogroups 1 and 2 captured similar profiles in terms of disease risk. To assess heterogeneity within LBBB ECGs more distinctly, we further investigated trends of disease risk within these ECGs only across the tree dimensions. We found that LBBB ECGs further along dimension 1 (toward the right region of the tree) were associated with greater risk of future ventricular arrythmia, AF, LVSD, and myocardial infarction but lower risk of complete heart block when covariate‐adjusted, including adjustment for QRS duration (Figure S11A), whereas LBBB ECGs further along dimension 2 were associated with lower risk of atherosclerotic cardiovascular disease, complete heart block, and LVSD. In predicted survival time plots (Figure S11B), these trends aligned closely with the predicted risk probabilities for LBBB ECGs in these dimensions. Overall, these results showed that LBBB ECGs within different regions of the tree capture distinct risk profiles for specific cardiovascular diseases, challenging the simplistic approach of treating LBBB as an homogenous group.

### Trends Across Echocardiography Measures

**Figure 8:**
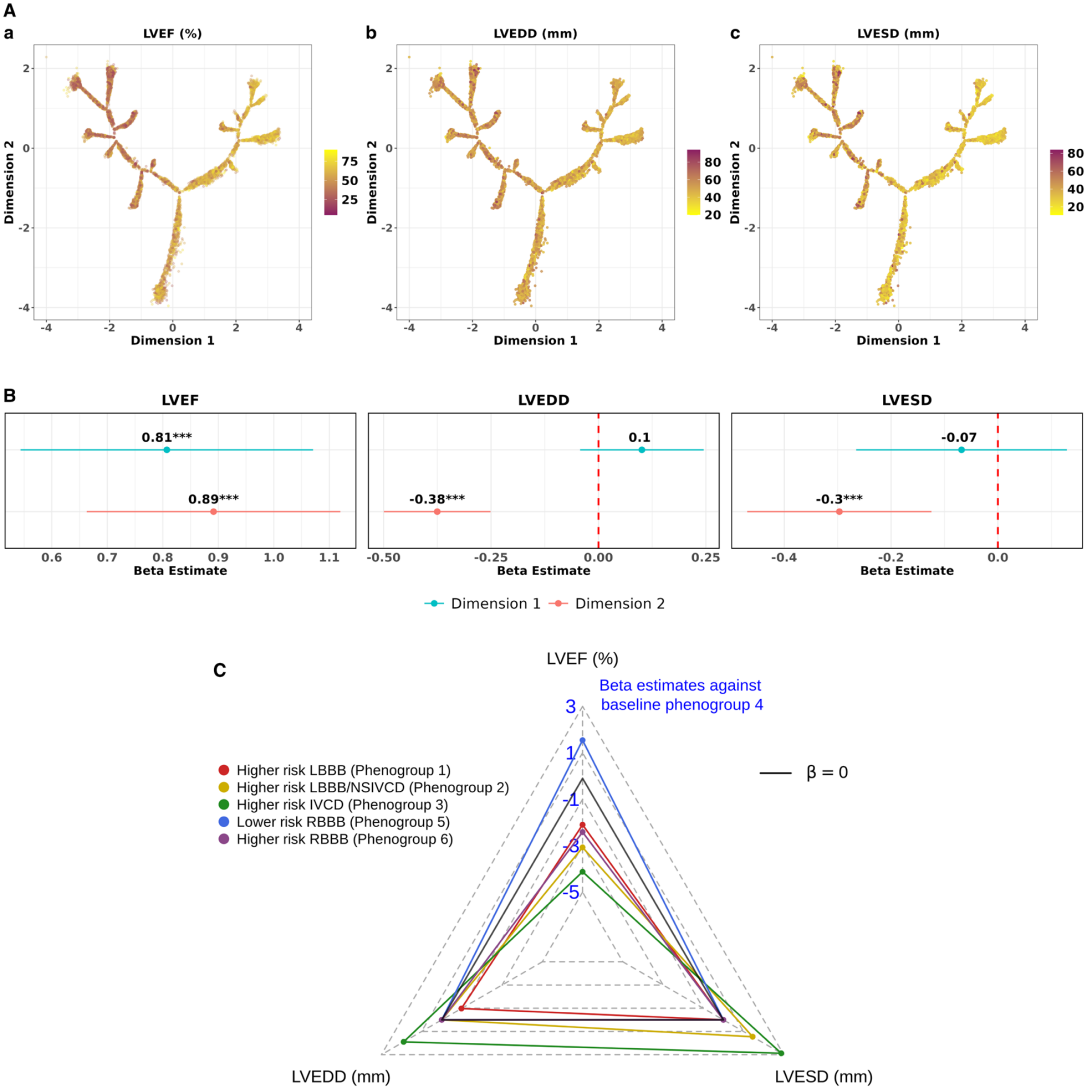
Trends across echocardiography measures in the broad QRS BIDMC DDRTree and its derived tree variables. **A**, Overlaying echocardiography measures (LVEF, LVEDD, LVESD) on the tree. **B**, Forest plots showing the adjusted beta estimates for tree dimensions (dimension 1 and 2) across the echocardiography end points. The red line refers to an adjusted β estimate of 0, indicating nonsignificance. All regression models presented were adjusted for age, sex, heart rate, QRS, and QTc interval and type of QRS morphology (right or left bundle‐branch block, nonspecific intraventricular conduction delay). **C**, Radar plot showing adjusted beta (β) estimates for each phenogroup across the different echocardiography measures against baseline comparator phenogroup 4. The regression estimates that did not reach statistical significance (*P* value >0.05) were set at a β of 0 in the plot. BIDMC indicates Beth Medical Israel Deaconess Center; DDRTree, dimensionality reduction via learning a tree; IVCD, intraventricular conduction delay, LBBB, left bundle‐branch block; LVEF, left ventricular ejection fraction; LVEDD, left ventricular end‐diastolic diameter; LVESD, left ventricular end‐systolic diameter; NSIVCD, nonspecific intraventricular conduction delay; and RBBB right bundle‐branch block.

A subset of BIDMC patients also had echocardiography recordings within 60 days of their ECGs available (12 381 ECGs, n=4723 unique patients). This was used to characterize differences in cardiac structure and function within the tree. We visualized trends across left ventricular echocardiography measures across the tree in Figure 8A. We assessed associations between echocardiography measures and the tree dimensions, adjusted for relevant covariates, in Figure 8B. Additionally, we quantified associations of phenogroup assignments against these end points, with full regression estimates in Table S13, Figure S12 and significant associations summarized in Figure 8C (non‐significant estimates indicated with beta estimate of 0).

Overlaying echocardiography measures on the tree indicated that lower values of LVEF and higher values of LVESD and LV end‐diastolic diameter were seen in the top‐left region (Figure 8A). But upon adjusting for covariates, both tree dimensions were associated with increasing LVEF whereas trends of decreasing LVEDD and LVESD were significant only along dimension 2 (Figure 8B). This suggested the top region of the tree associated with more normal echocardiography measures (increasing LVEF, decreasing LVEDD and LVESD) and the top‐right region was distinctly associated with improving LVEF, when accounting for the effect of covariates. Correspondingly, the lower risk RBBB phenogroup was associated to a significant increase in LVEF with a beta estimate of 1.54 (0.50–2.59, *P*<0.01), compared with phenogroup 4 (Figure 8C). Overall, the higher risk NSIVCD‐dominant phenogroup had the most abnormal echocardiography profile, with a 4.12 (−5.54 to −2.71, *P*<0.0001) decrease per unit in LVEF, a 1.90 (1.12–2.67, *P*<0.0001) increase per unit in LVEDD, and a 2.88 (1.81–3.95, *P*<0.0001) increase per unit in LVESD than phenogroup 4.

### Patterns in Cardiac Resynchronization Therapy Response

Lastly, we investigated how CRT response varied across the tree using ECGs linked echocardiography measurements taken before and 6 months post CRT. This was done in 2 distinct cohorts, a subset of participants from BIDMC (443 ECGs, n=110) and a separate external data set from a CRT study (n=141) conducted at UVA. As CRT is currently typically used in the context of LBBB, we restricted our analysis to these subjects in both cohorts. The UVA cohort was treated as an independent test set, and the BIDMC cohort served as the training set.

**Figure 9:**
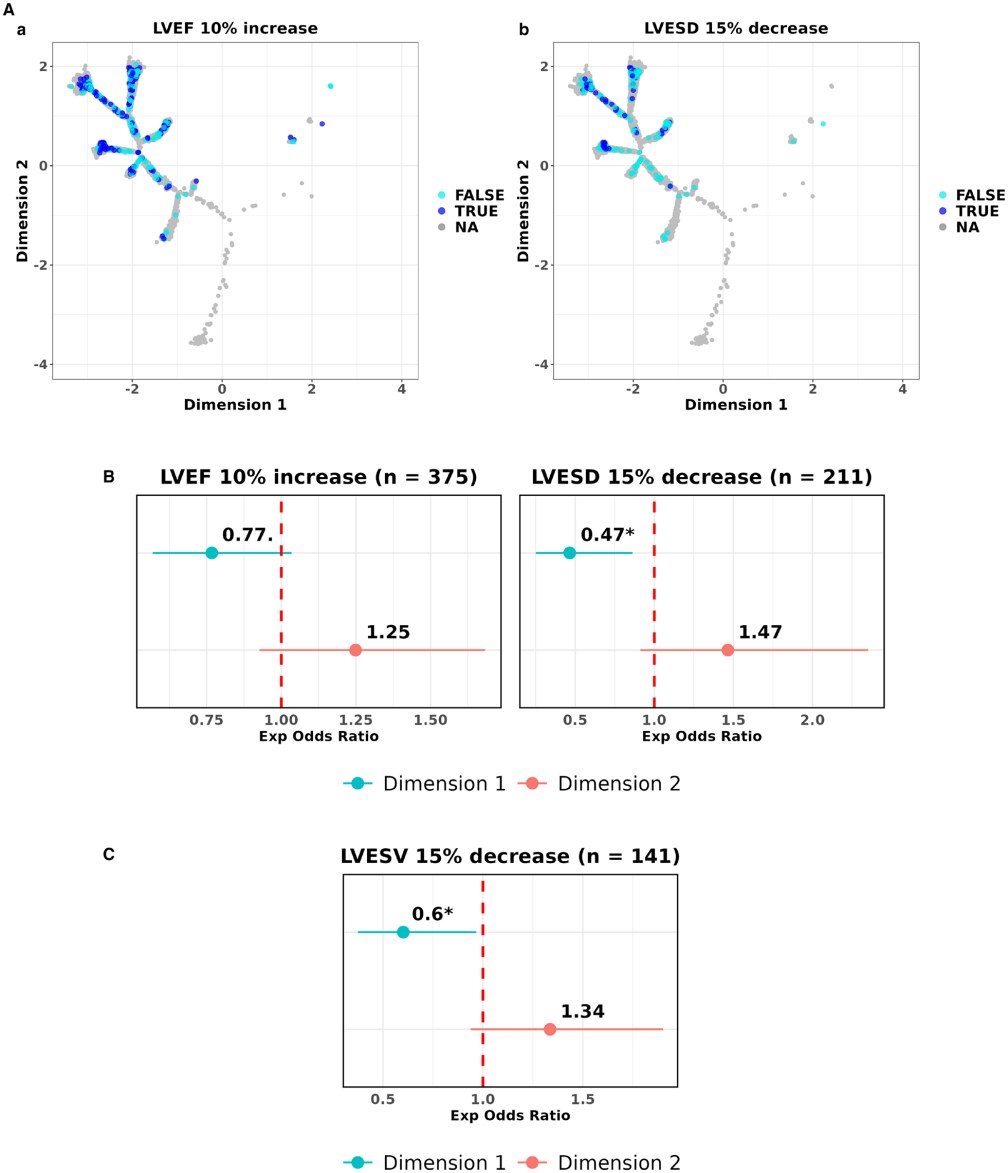
Assessing trends in cardiac resynchronization therapy responses across LBBB ECGs. **A**, Two CRT response end points were visualized across LBBB ECGs within the BIDMC DDRTree: LVEF 10% increase and LVESD 15% decrease. Dark and light blue instances indicate whether CRT response was observed or not respectively, in each end point. Gray instances refer to ECGs linked to echocardiography instances with missing values for LVEF or LVESD either before or 6 months post CRT. **B**, Forest plots showing the adjusted exponentiated odds ratios for tree dimensions (dimensions 1 and 2) against CRT response end points in the BIDMC cohort. **C**, Forest plot showing the adjusted exponentiated odds ratios for tree dimensions (dimensions 1 and 2) against the LVESV 15% decrease CRT response end point in the UVA cohort. The red line refers to an exponentiated odds ratio of 1, indicating nonsignificance. All regression models presented were adjusted for age, sex, and QRS duration. BIDMC indicates Beth Medical Israel Deaconess Center; CRT, cardiac resynchronization therapy; DDRTree, dimensionality reduction via learning a tree; LBBB; left bundle‐branch block; LVEF, left ventricular ejection fraction; LVESD, left ventricular end‐systolic diameter; LVESV, left ventricular end‐systolic volume; and UVA, University of Virginia.

The LVEF 10% increase and LVESD 15% decrease end points were assessed in the BIDMC cohort and the LVESV 15% decrease end point was assessed in the UVA cohort based on data availability and missingness. For the BIDMC cohort, we visualized CRT responses across the tree (Figure 9A). Additionally, we quantified whether the tree dimensions captured relevant trends of CRT response across the 3 end points (Figure 9B). To understand the influence of ECG position within LBBB phenogroups on CRT response, additional analyses were done, restricted to the BIDMC cohort due to more data points being available (Table S14).

Moving along dimension 1 (from left to right of the tree) was significantly associated with 53% lower odds (OR, 0.47 [95% CI, 0.25–0.86], *P*<0.05) of CRT response by LVESD criteria whereas dimension 2 did not have any association with both CRT response end points (Figure 9B).

Similarly, in the UVA cohort, moving along dimension 1 was significantly associated with 40% lower odds (OR, 0.60 [95% CI, 0.37–0.97], *P*<0.05) for the LVESV criteria whereas dimension 2 did not achieve statistical significance, when adjusted for QRS duration (Figure 9C). Additionally, in the BIDMC DDRTree, moving within the higher risk LBBB‐NSIVCD phenogroup 2 toward its branch periphery was associated with significantly increased odds of CRT response by the LVEF criteria, when adjusted, suggesting position within this phenogroup captured CRT response distinctly (Table S14, Figure S13). Similar trends were also observed for the CRT response relating to LVESD decrease but these associations did not achieve statistical significance. These results imply that ECGs within the LBBB‐dominant BIDMC tree region capture crucial traits influencing CRT response. Furthermore, the significance of positioning an ECG within the branch on CRT response differed between the 2 LBBB phenogroups.

## DISCUSSION

Using an unsupervised machine learning approach, we modeled the various broad QRS phenotypes as a data‐driven taxonomy and identified 6 phenogroups of abnormal ventricular depolarization, with the aim of disambiguating more precise pathophysiology obscured by traditional taxonomy. The identified phenogroups have similarities with the existing paradigm of LBBB and RBBB but go beyond these simplistic historical classifications. Although the DDRTree‐derived phenogroups showed a modest improvement in the *c*‐statistic for time‐to‐death prediction compared with the QRS morphology‐derived phenogroups (0.705 versus 0.701), this difference may not be clinically meaningful.

However, our analysis reveals important and clinically relevant differences within LBBB and within RBBB, highlighting subgroups at high risk of disease and provide additive value in predicting CRT response. Moreover, we extracted valuable insights by examining how these phenogroups are arranged in a low dimensional 2‐dimensional space and identifying trends across the tree region. This underscores the limitations of other clustering methods that do not consider the meaningful placement of clusters, and subjects within clusters.

Although traditional classification methods offer practical benefits for initial clinical assessment, they overlook the relevant heterogeneity within broad QRS subtypes. Using a continuous approach is expected to provide more accurate results, offering greater granularity and prompting research into incorporating a precision medicine viewpoint into current clinical approaches that balance biological relevance with practical usability.

### LBBB‐Dominant Phenogroups

To the best of our knowledge, this is the first study to investigate underlying phenogroups within a population of individuals with broad QRS durations. Our findings in the LBBB‐dominant phenogroups are consistent with prior descriptions, lending credence to the biological relevance of DDRTree defined phenogroups. In particular, individuals with LBBB are more likely to be female^26^ and have a greater risk of HF,^27^ which is often due to reduced EF.^28^ In patients with HF, presence of LBBB is well established to attribute to increased mortality risk and hospitalization risk.^29^, ^30^ The LBBB‐dominant phenogroups 1 and 2 show similar, overlapping traits of high‐risk LBBB likely influenced by phenogroup 1 emerging from the periphery of phenogroup 2. However, we were able to demonstrate how DDRTree dimensions captured variation within a LBBB subpopulation with variability in CRT response, even after correcting for QRS duration and was retained when validated in a separate, external CRT data set. LBBB ECGs located in tree regions with greater density of RBBB ECGs (moving from left to right of the tree) associated with lower likelihood of CRT response and greater risk of disease. Interestingly, the incidence of complete heart block was reduced toward the right of the tree (along dimension 1), in contrast to other adverse events including ventricular arrythmia that were increased in that direction. This may suggest that the tree captures, in part, the distinction between proximal conduction system disease (which would lead to complete heart block) and myocardial conduction disease (which may predispose to ventricular arrythmia). This may also be helpful in distinguishing between patients who would benefit from conduction system pacing (the former group) and those who will not (the latter group).

Studies that have used unsupervised machine learning to phenogroup CRT responders have shown similar trends where phenogroups that associated with greater CRT nonresponse also associated with greater risk of death, cardiovascular death, and future HF hospitalization.^31^, ^32^ Our observations challenge the conventional view of LBBB as a distinct risk category without gradation. Although LBBB does pose greater risk than RBBB, our findings suggest a nuanced impact on adverse events. Like LBBB, NSIVCD‐dominant phenogroup 3 was also associated with high risk. Individuals with IVCD and other cardiovascular disease burden have been associated to increased mortality risk^33^ as well as arrhythmic death when reduced EF was also observed.^34^

### RBBB‐Dominant Phenogroups

In contrast, more distinct differences within the RBBB‐dominant phenogroups were observed regarding cardiovascular disease burden and future disease risk, and trends for baseline traits were followed; men were more frequently represented in these phenogroups.^35^ Specifically, there was an indication of isolated RBBB within phenogroup 5 and a high‐risk RBBB subtype within phenogroup 6. An interesting finding was greater all‐cause and cardiovascular mortality risk being experienced by the high‐risk group with RBBB. Although RBBB in individuals with prevalent cardiovascular morbidities has been associated with increased risk of mortality,^36^, ^37^ traditionally LBBB has been associated with greater risk than RBBB in a diseased population.^38^, ^39^ Historically, isolated RBBB was thought not to increase cardiac risk,^40^, ^41^ based on findings from small patient cohorts. However, recent studies present conflicting findings.^42^ Whereas some suggest a link between isolated RBBB and mortality risk^36^ or progression to high‐degree atrioventricular block,^43^ others indicate increased risk only when combined with left anterior or posterior fascicular block, leading to HF^28^, ^44^ and mortality.^45^, ^46^ Our findings help reconcile the prior conflicting evidence on RBBB and suggest both high‐risk and low‐risk RBBB phenogroups exist.

### Clinical Implications

ECGs are routinely used in the clinical evaluation of patients with cardiac conditions to assess their prognosis and repeated when there is clinical indication or suspicion of other cardiac abnormalities. By using these ECGs to project patients onto an established DDRTree capturing key traits within a diseased population, they can be mapped to specific phenogroups. Over time their ECGs can also be used to map their progression across the tree and understand their changes in disease progression. One use case would be to identify risk of future HF or LVSD in patients with broad QRS. Subjects at low risk of future HF may be reassured, and higher risk individuals who are progressing along the tree over time could have intermittent screening echocardiography or even preemptive HF therapies. Additionally, our tree allows improved prediction of which subjects will be CRT responders, this may help improve patient selection for CRT and more specifically, conduction system pacing. Similarly, existence of high‐risk groups with RBBB challenges the current paradigm of RBBB being viewed to be low risk. Subjects in the high‐risk RBBB phenogroup may benefit from further investigation including echocardiography and serial follow‐up. The increasing prospects of using conduction system pacing as a new CRT modality^47^ has generated renewed interest in differentiating LBBB from IVCD because only true blocks in LBBB can be unblocked by conduction system pacing. Hence this has intensified the importance of classifying broad QRS complexes into biological relevant categories by considering morphological features that capture underlying physiology. Future work would include incorporating morphological features within the tree to delineate these phenogroups further.

### Limitations

This study had several limitations. To increase the number of ECGs for a comprehensive broad QRS DDRTree, multiple ECGs per patient from the BIDMC cohort were used, which may lead to overrepresentation of certain subjects within the tree. QRS morphology labels for the BIDMC non‐CRT subset and the external validation UKB cohort were predicted using a deep neural network model,^23^ as previously mentioned, using 10‐second ECGs as input rather than assigned by clinicians. Although in validation studies this model performance was superior to clinicians, there is a possibility of misclassification errors. Furthermore, the model predicted binary labels of RBBB and LBBB, so NSIVCD labels used in this analysis were interpreted in scenarios where neither BBB was observed. In the CRT analyses, we were limited to adjusting for confounders such as age, sex, and QRS duration due to the small sample size of the CRT subsets within the cohorts investigated. Clustering approaches depend on the underlying patient population, and derived clusters may vary with population characteristics. Although we demonstrated consistent phenogroups across independent broad QRS populations in this work, this dependency should be considered applying clustering methods to other populations. Lastly, we were unable to offer recommendations for human ECG readers to visually subcategorize QRS types into the derived phenogroups, which is essential for clinical implementation. This underscores the main challenge addressed by this study: machine learning models can detect subtle ECG features, indistinguishable to the human eye, that differentiate phenogroups.

## CONCLUSIONS

Using a data‐driven taxonomy, we have demonstrated that the classical categorization of QRS morphology should be refined to consider subgroups that exist within them. These subgroups differ substantially in future risk of disease and response to CRT and may be used to guide investigations, follow‐up, and intervention strategies in patients with a broad QRS complex.

## Sources of Funding

Arunashis Sau is funded by a British Heart Foundation clinical research training fellowship (FS/CRTF/21/24183). Fu Siong Ng, Nicholas S. Peters, Arunashis Sau, Lara Curran, and Mehak Gurnani are supported by the British Heart Foundation (RG/F/22/110078 and RE/18/4/34215). Fu Siong Ng and Declan O'Regan are supported by the National Institute for Health Research Imperial College Biomedical Research Centre. Ewa Sieliwonczyk is supported by a European Joint Programme on Rare Diseases Research Mobility Fellowship (European Reference Networks). Declan O'Regan is supported by the Medical Research Council (MC_UP_1605/13, MC‐A658‐5TY00) and the British Heart Foundation (RG/19/6/34387, RE/18/4/34215, CH/P/23/80008).

## Disclosures

Jonathan W. Waks and Daniel B. Kramer were previously on the advisory board for Heartcor solutions LLC, for whom they remain independent consultants. The remaining authors have no disclosures to report.

## Supporting information

Data S1
Tables S1–S14
Figures S1–S13
Reference 48
