# Supplementary material for: Revisiting Abnormalities of Ventricular Depolarization: Redefining Phenotypes and Associated Outcomes Using Tree‐Based Dimensionality Reduction
Source: J Am Heart Assoc. 2025 Jun 18;14(13):e040814. doi: 10.1161/JAHA.124.040814 (PMC12449969; doi:10.1161/JAHA.124.040814)
Supplement: Supplementary file 1 — Data S1 Tables S1–S14 Figures S1–S13 Reference 48 [file JAH3-14-e040814-s001.pdf]

Supplementary Methods (Data S1)

Merging DDRTree-derived branches using hierarchical clustering

Sub-branches returned by DDRTree were merged using an unsupervised machine learning method, hierarchical clustering. Clusters were defined based on proximity between sub-branches hence utilised the normalised tree coordinates of sub-branches as input to the model. Euclidean distance was used to estimate distances between pairs of points with the “average” linkage criterion. From the resulting dendrogram, average silhouette width and silhouette width within the clusters at different dendrogram heights were used to determine the ideal number of clusters. The ideal number of clusters was determined to be six, balancing the need for similar distributions within individual clusters while maximising the overall silhouette score. The overall average silhouette score across the six clusters was 0.45.

Variational Autoencoder: model training and hyperparameters

The VAE was trained as an annealed variational autoencoder[48], using the BIDMC dataset and a 85/5/10% training/validation/test split (grouped by patient ID). The reconstruction loss was set as the mean absolute error and the Kullback-Leibler loss was weighted with a beta of 10, which ensured maximal latent feature disentanglement. The model was trained for 300 epochs using the Adam optimizer, a batch size of 128, and with a learning rate of 0.0005. The number of latent features (51) was determined by the number of latent units with information capacity above 0.1 (as defined by KL divergence), which captured all meaningful variation in the data.

Tuning Elastic net parameters for explainability analyses

We used Elastic Net regularised multivariate logistic regression to analyse how the latent features associated with the derived phenogroups. The model tuning involved optimising two parameters: alpha (which controls the balance between L1 and L2 regularisation) and lambda

(which controls the regularisation strength). We applied 5-fold cross-validation, minimising log-loss, to determine the optimal alpha value. After tuning alpha, we fitted glmnet models to optimise the lambda value, again minimising log-loss. The final model was selected based on the coefficients at the lambda.1se value (the lambda value at 1 standard error from the minimum cross-validation error), ensuring the most regularised model was chosen. These steps were applied independently to each phenogroup to find the best-fitting model for each. The log-odds coefficients were ordered by their absolute values to identify the top 3 predictors for each phenogroup, and the latent patterns of these features were visualized.

Table S1

Demographic characteristics of the non-CRT cohorts included in this study. Data are shown for the broad QRS populations within the BIDMC non-CRT subset and the UK Biobank (UKB). Continuous variables are shown as median values with the associated interquartile ranges (25th-75th percentile) and categorical variables are shown with their frequencies.

	**BIDMC (non-CRT subset)**	**UK Biobank**
**Number of ECGs**	41,242	2,319
**Number of unique subjects**	11,000	2,319
**Demographics**		
Age, years	73 (65–81)	71 (65–75)
Sex (female)	13,789 (33%)	667 (29%)
BMI	28 (25–32)	26.7 (24.3–29.8)
Systolic BP, mm Hg	131 (122–142)	148 (136–163)
QRS duration (ms)	140 (130–152)	136 (128–146)
**QRS morphology**		
RBBB	21,388 (52%)	1,334 (58%)
LBBB	10,759 (26%)	733 (32%)
NSIVCD	9,095 (22%)	252 (11%)
**All-cause mortality**	13,201 (32%)	45 (1.9%)
**Cardiovascular death**	4,916 (12%)	-
**Heart Failure**	18,341 (44%)	79 (3.4%)
**Myocardial Infarction**	11,578 (28%)	147 (6.3%)
**Atherosclerotic CVD**	27,129 (66%)	-
**EF ≤ 50%**	10,945 (27%)	-
**Atrial Fibrillation**	18,669 (45%)	169 (7.3%)
**Ventricular Arrythmia**	4,769 (12%)	-

**Hypertension**	33,884 (82%)	2 (<0.1%)
Values are expressed as median (interquartile range 25th-75th percentile) or n (%)<br><br>*BIDMC Beth Israel Deaconess Medical Centre; BMI body mass index; BP blood pressure; CRT cardiac resyncronisation therapy; CVD cardiovascular disease; EF ≤ 50% impaired left ventricular function (ejection fraction < 50%); LBBB left bundle branch block; NSIVCD non-specific intraventricular conduction delay; RBBB right bundle branch block; UKB UK Biobank.*		

Table S2

Demographic characteristics of the CRT cohorts included in this study. Data are shown for the broad QRS populations within the BIDMC CRT subset and the University of Virginia cohort (UVA). Continuous variables are shown as median values with the associated interquartile ranges (25th-75th percentile) and categorical variables are shown with their frequencies.

	**BIDMC (CRT subset)**	**UVA**
**Number of ECGs**	1,296	141
**Number of unique subjects**	150	141
**Demographics**		
Age, years	69 (60–76)	66.4 (57.0-72.4)
Sex (female)	370 (29%)	46 (32.6%)
BMI	30.8 (26.4–33.8)	29.5 (25.8-34.4)
Systolic BP, mm Hg	121 (113–134)	118.0 (105.0-130.0)
QRS duration (ms)	152 (140–164)	155.0 (140.0-169.0)
**QRS morphology**		
RBBB	55 (4.3%)	0
LBBB	1,132 (88%)	0
NSIVCD	97 (7.6%)	141 (100%)
**All-cause mortality**	368 (28%)	119 (84.4%)
**Cardiovascular death**	208 (16%)	-
**Heart Failure**	953 (74%)	-
**Myocardial Infarction**	393 (30%)	-
**Atherosclerotic CVD**	789 (61%)	-
**EF ≤ 50%**	888 (69%)	-
**Atrial Fibrillation**	562 (43%)	29 (20.6%)
**Ventricular Arrythmia**	131 (10%)	-
**Hypertension**	977 (75%)	81 (57.4%)

Values are expressed as median (interquartile range 25th-75th percentile) or n (%)

BIDMC Beth Israel Deaconess Medical Centre; BMI body mass index; BP blood pressure; CRT cardiac resyncronisation therapy; CVD cardiovascular disease; EF ≤ 50% impaired left ventricular function (ejection fraction < 50%); LBBB left bundle branch block; NSIVCD non-specific intraventricular conduction delay; RBBB right bundle branch block; UVA = University of Virginia.

Table S3

Descriptive statistics of the broad QRS DDRTree phenogroups in the BIDMC population across baseline variables, ECG measures, QRS morphology, echocardiography parameters, cardiac resynchronization therapy (CRT) response variables and cardiovascular disease and mortality outcomes. Continuous variables are shown as median values with the associated interquartile ranges (25th-75th percentile) and categorical variables are shown with their frequencies. Differences across phenogroups were quantified using the non-parametric Kruskal-Wallis rank sum test and Pearson's Chi-squared test for continuous and categorical variables, respectively using a p-value significance threshold of 0.05. Unknown values refer to missing values for variables.

BIDMC Beth Medical Isreal Deaconess Centre.

Variable	Phenogroup 1, N = 5,902*	Phenogroup 2, N = 8,917*	Phenogroup 3, N = 6,145*	Phenogroup 4, N = 4,863*	Phenogroup 5, N = 12,123*	Phenogroup 6, N = 4,588*	p-value**
**Baseline variables**							
**Age**	73 (65–82)	74 (65–81)	72 (63–79)	73 (65–81)	73 (65–80)	74 (66–82)	<0.001
**Male**	2,859 (48%)	5,421 (61%)	4,689 (76%)	3,440 (71%)	8,568 (71%)	3,402 (74%)	<0.001
**Ethnicity**							
Asian	170 (2.9%)	183 (2.1%)	144 (2.3%)	194 (4.0%)	592 (4.9%)	158 (3.4%)	
Black	614 (10%)	767 (8.6%)	556 (9.0%)	292 (6.0%)	850 (7.0%)	369 (8.0%)	
Caribbean	0 (0%)	1 (<0.1%)	1 (<0.1%)	2 (<0.1%)	1 (<0.1%)	2 (<0.1%)	
Hispanic	427 (7.2%)	408 (4.6%)	229 (3.7%)	256 (5.3%)	459 (3.8%)	188 (4.1%)	
Indigenous/Pacific Islander	18 (0.3%)	25 (0.3%)	10 (0.2%)	10 (0.2%)	5 (<0.1%)	7 (0.2%)	
Unknown	331 (5.6%)	462 (5.2%)	294 (4.8%)	219 (4.5%)	598 (4.9%)	198 (4.3%)	
White	4,342 (74%)	7,071 (79%)	4,911 (80%)	3,890 (80%)	9,618 (79%)	3,666 (80%)	
**BMI**	28 (25–32)	28 (25–32)	29 (26–33)	29 (26–33)	28 (25–32)	29 (26–33)	<0.001
Unknown	662	1,186	1,041	916	2,510	686	

**Systolic Blood Pressure (mmHg)**	132 (121–143)	130 (120–141)	128 (119–139)	132 (123–142)	133 (124–143)	129 (120–139)	<0.001
Unknown	608	1,153	871	512	1,581	481	
**Diastolic Blood Pressure (mmHg)**	70 (64–77)	71 (65–78)	72 (65–79)	72 (66–78)	72 (66–79)	71 (65–77)	<0.001
Unknown	611	1,153	872	512	1,584	482	
**Non-ischaemic cardiomyopathy**	341 (71%)	356 (60%)	72 (49%)	7 (78%)	29 (83%)	9 (28%)	
Unknown	5,422	8,325	5,997	4,854	12,088	4,556	
**ECG measures**							
**Heart Rate**	66 (59–76)	69 (60–79)	68 (60–78)	68 (60–79)	65 (57–74)	70 (61–81)	<0.001
**QRS interval**	148 (138–160)	138 (128–150)	130 (124–144)	140 (130–148)	140 (132–150)	144 (134–154)	<0.001
**PR interval**	178 (160–202)	180 (160–206)	180 (156–208)	176 (154–200)	172 (152–194)	178 (158–203)	<0.001
Unknown	921	2,077	1,747	821	1,816	955	
**QTc interval**	476 (456–496)	468 (446–490)	461 (438–485)	459 (439–481)	455 (436–477)	468 (446–490)	<0.001
**QRS Axis**							
**Normal QRS Axis**	1,268 (38%)	2,602 (44%)	1,716 (39%)	1,318 (37%)	4,698 (60%)	523 (15%)	<0.001
Unknown	2,587	3,055	1,731	1,324	4,313	1,112	
**Right Axis Deviation**	14 (0.4%)	78 (1.3%)	172 (3.9%)	276 (7.8%)	692 (8.9%)	488 (14%)	<0.001
Unknown	2,587	3,055	1,731	1,324	4,313	1,112	
**Left Axis Deviation**	2,010 (61%)	3,095 (53%)	2,251 (51%)	1,689 (48%)	2,200 (28%)	1,896 (55%)	<0.001
Unknown	2,587	3,055	1,731	1,324	4,313	1,112	
**Extreme Axis Deviation**	23 (0.7%)	87 (1.5%)	275 (6.2%)	256 (7.2%)	220 (2.8%)	569 (16%)	<0.001
Unknown	2,587	3,055	1,731	1,324	4,313	1,112	
**QRS Morphology**							<0.001
NSIVCD	751 (13%)	3,072 (34%)	3,990 (65%)	340 (7.0%)	598 (4.9%)	441 (9.6%)	
LBBB	5,145 (87%)	5,740 (64%)	913 (15%)	6 (0.1%)	29 (0.2%)	58 (1.3%)	
RBBB	4 (<0.1%)	102 (1.1%)	1,237 (20%)	4,515 (93%)	11,496 (95%)	4,089 (89%)	
Unknown	2	3	5	2	0	0	

**Echocardiography measures**							
**LVEF (%)**	46 (32–59)	48 (32–60)	53 (35–60)	60 (52–68)	60 (55–70)	57 (43–65)	<0.001
Unknown	3,894	6,058	4,481	3,694	9,430	3,325	
**LVEDD (mm)**	49 (43–56)	50 (44–57)	52 (46–58)	47 (42–52)	47 (42–52)	48 (42–54)	<0.001
Unknown	3,980	6,294	4,689	3,789	9,629	3,460	
**LVESD (mm)**	35 (29–45)	36 (29–46)	36 (30–44)	30 (25–35)	30 (26–34)	31 (25–37)	<0.001
Unknown	4,656	7,187	5,144	4,046	10,184	3,794	
**CRT Response Variables**							
**LVEF 10% increase**	241 (60%)	266 (56%)	31 (25%)	1 (20%)	6 (19%)	8 (26%)	
Unknown	5,498	8,438	6,019	4,858	12,092	4,557	
**LVESD 15% decrease**	80 (42%)	108 (45%)	14 (19%)	0 (0%)	3 (60%)	0 (0%)	<0.001
Unknown	5,710	8,676	6,070	4,862	12,118	4,568	
**Mortality outcomes**							
**All cause mortality**	2,045 (35%)	3,052 (34%)	2,007 (33%)	1,486 (31%)	3,232 (27%)	1,747 (38%)	<0.001
**Cardiovascular Death**	827 (14%)	1,168 (13%)	781 (13%)	541 (11%)	1,054 (8.7%)	753 (16%)	<0.001
**Prevalent disease**							
**Heart Failure**	3,571 (61%)	5,059 (57%)	2,989 (49%)	1,817 (37%)	3,654 (30%)	2,204 (48%)	<0.001
**Myocardial Infarction**	1,642 (28%)	2,773 (31%)	1,987 (32%)	1,354 (28%)	2,677 (22%)	1,538 (34%)	<0.001
**Atherosclerotic CVD**	4,069 (69%)	6,046 (68%)	3,990 (65%)	3,222 (66%)	7,363 (61%)	3,228 (70%)	<0.001
**EF < 50%**	2,517 (43%)	3,560 (40%)	2,074 (34%)	870 (18%)	1,530 (13%)	1,282 (28%)	<0.001
**Atrial Fibrillation**	2,409 (41%)	4,309 (48%)	3,396 (55%)	1,925 (40%)	4,944 (41%)	2,248 (49%)	<0.001
**Ventricular Arrhythmia**	611 (10%)	1,165 (13%)	1,058 (17%)	449 (9.2%)	1,028 (8.5%)	589 (13%)	<0.001
**Hypertension**	4,902 (83%)	7,333 (82%)	4,808 (78%)	4,099 (84%)	9,857 (81%)	3,862 (84%)	<0.001
**Type 2 Diabetes Mellitus**	1,136 (19%)	1,589 (18%)	987 (16%)	995 (20%)	1,865 (15%)	1,092 (24%)	<0.001

* Median (25%–75%); n (%)

** Kruskal-Wallis rank sum test; Pearson's Chi-squared test; Fisher's exact test

BMI body mass index; CRT cardiac resynchronisation therapy; EF ≤ 50% impaired left ventricular function (ejection fraction < 50%); LBBB left bundle branch block; LVEF left ventricular ejection fraction; LVEDD left ventricular end-diastolic diameter; LVESD left ventricular end-systolic diameter; NSIVCD non-specific intraventricular conduction delay; RBBB right bundle branch block.

Table S4

Descriptive statistics of the broad QRS DDRTree phenogroups in the UK Biobank population across baseline variables, ECG measures, QRS morphology and available cardiovascular disease and mortality outcomes. Continuous variables are shown as median values with the associated interquartile ranges (25th-75th percentile) and categorical variables are shown with their frequencies. Differences across phenogroups were quantified using the non-parametric Kruskal-Wallis rank sum test and Pearson's Chi-squared test for continuous and categorical variables, respectively using a p-value significance threshold of 0.05. Unknown values refer to missing values for variables.

Variable	Phenogroup 1, N = 453*	Phenogroup 2, N = 339*	Phenogroup 3, N = 170*	Phenogroup 4, N = 272*	Phenogroup 5, N = 961*	Phenogroup 6, N = 124*	p-value**
**Baseline variables**							
**Age**	71 (65–75)	71 (66–75)	70 (65–75)	71 (66–76)	71 (65–75)	72 (67–76)	0.18
**Male**	227 (50%)	211 (62%)	134 (79%)	222 (82%)	757 (79%)	101 (81%)	<0.001
**BMI**	26.6 (24.3–29.8)	26.1 (23.7–29.0)	26.7 (24.4–30.6)	27.5 (24.9–30.1)	26.7 (24.4–29.5)	28.1 (25.0–30.9)	<0.001
Unknown	12	7	6	4	24	3	
**Systolic Blood Pressure (mmHg)**	149 (137–164)	150 (136–164)	147 (135–163)	148 (136–161)	148 (136–162)	150 (141–164)	0.47
Unknown	105	71	45	64	201	27	
**Diastolic Blood Pressure (mmHg)**	78 (71–85)	77 (70–84)	80 (71–88)	79 (73–86)	80 (72–87)	79 (69–85)	0.008
Unknown	104	71	45	64	201	27	
**ECG measures**							
**Heart Rate**	61 (55–67)	60 (54–68)	59 (53–68)	63 (56–71)	61 (55–68)	65 (55–71)	0.008
Unknown	156	131	56	92	315	43	
**QRS interval**	144 (136–156)	134 (126–144)	130 (126–142)	134 (128–142)	136 (128–144)	138 (128–148)	<0.001
**PR interval**	174 (160–192)	176 (158–198)	172 (152–202)	171 (156–200)	172 (154–188)	176 (150–212)	0.27

Unknown	180	148	73	114	365	55	
**QTc interval**	462 (447–480)	453 (436–470)	444 (423–467)	447 (429–464)	448 (431–465)	448 (430–467)	<0.001
Unknown	156	131	56	92	315	43	
**QRS Axis**							
**Normal QRS Axis**	198 (67%)	142 (68%)	42 (37%)	103 (57%)	501 (78%)	21 (26%)	<0.001
Unknown	156	131	56	92	315	43	
**Right Axis Deviation**	1 (0.3%)	3 (1.4%)	9 (7.9%)	15 (8.3%)	40 (6.2%)	7 (8.6%)	
Unknown	156	131	56	92	315	43	
**Left Axis Deviation**	98 (33%)	63 (30%)	60 (53%)	60 (33%)	100 (15%)	52 (64%)	<0.001
Unknown	156	131	56	92	315	43	
**Extreme Axis Deviation**	0 (0%)	0 (0%)	0 (0%)	0 (0%)	0 (0%)	0 (0%)	
Unknown	156	131	56	92	315	43	
**QRS Morphology**							
**LBBB**	437 (96%)	266 (78%)	28 (16%)	0 (0%)	0 (0%)	2 (1.6%)	<0.001
**RBBB**	0 (0%)	8 (2.4%)	42 (25%)	243 (89%)	930 (97%)	111 (90%)	<0.001
**NSIVCD**	16 (3.5%)	65 (19%)	100 (59%)	29 (11%)	31 (3.2%)	11 (8.9%)	<0.001
**Mortality outcomes**							
**All-cause mortality**	9 (2.0%)	8 (2.4%)	3 (1.8%)	4 (1.5%)	17 (1.8%)	4 (3.2%)	0.83
**Prevalent disease**							
**Heart Failure**	26 (5.7%)	24 (7.1%)	7 (4.1%)	3 (1.1%)	15 (1.6%)	4 (3.2%)	
**Myocardial Infarction**	27 (6.0%)	32 (9.4%)	15 (8.8%)	20 (7.4%)	38 (4.0%)	15 (12%)	<0.001
**Atrial Fibrillation**	22 (4.9%)	34 (10%)	11 (6.5%)	20 (7.4%)	70 (7.3%)	12 (9.7%)	0.11
**Hypertension**	2 (0.4%)	0 (0%)	0 (0%)	0 (0%)	0 (0%)	0 (0%)	0.24
* Median (25%–75%); n (%)							
** Kruskal-Wallis rank sum test; Pearson’s Chi-squared test; Fisher’s exact test							

BMI body mass index; LBBB left bundle branch block; NSIVCD non-specific intraventricular conduction delay; RBBB right bundle branch block.

Table S5

Frequency of incident events across the BIDMC (Beth Medical Isreal Deaconess Centre) cohort.

Outcome	Event Rate (%)
Ventricular arrythmia	8.35
Heart failure	29.79
Atrial fibrillation	20.93
Ejection fraction ≤ 50%	15.75
Atherosclerotic cardiovascular disease	28.78
Myocardial infarction	13.53
Cardiovascular death	12.05
Complete Heart Block	8.63
All-cause mortality	31.90

Table S6

Adjusted odds ratios from multivariate logistic models evaluating the association between phenogroup assignments and prevalent cardiovascular disease outcomes in the BIDMC broad QRS DDRTree population. Exponentiated odds ratios, 95% confidence intervals and associated p-values (corrected for multiple testing, p-value x 6) are presented for each phenogroup, against the baseline comparator RBBB phenogroup 4, adjusted for covariates. Covariates included age, sex, ECG measurements (heart rate, QRS duration, QTc interval) and type of QRS morphology (left or right bundle branch block (LBBB/RBBB) or non-specific intraventricular conduction delay (NSIVCD)).

	**Exponentiated Odds Ratio (95% confidence intervals) p-value**					
**Phenogroup**	**VA**	**HF**	**EF ≤ 50%**	**ASCVD**	**MI**	**AF**
LBBB Phenogroup 1	**0.61 (0.51-0.73), p < 0.0001**	**1.20 (1.06-1.36), p < 0.05**	**1.31 (1.14-1.50), p < 0.001**	1.06 (0.93-1.21), 2.12	0.86 (0.75-0.98), 0.16	0.93 (0.82-1.05), 1.49
LBBB-NSIVCD Phenogroup 2	0.81 (0.69-0.95), 0.06	**1.22 (1.09-1.37), p < 0.01**	**1.44 (1.27-1.64), p < 0.0001**	1.05 (0.94-1.18), 2.29	1.03 (0.91-1.16), 3.92	**1.18 (1.06-1.32), p < 0.05**
IVCD Phenogroup 3	1.11 (0.96-1.28), 1.02	**1.20 (1.08-1.32), p < 0.01**	**1.62 (1.45-1.82), p < 0.0001**	1.02 (0.92-1.13), 4.57	1.12 (1.01-1.25), 0.19	**1.43 (1.30-1.58), p < 0.0001**
RBBB Phenogroup 5	0.90 (0.80-1.02), 0.57	**0.73 (0.68-0.79), p < 0.0001**	**0.66 (0.61-0.73), p < 0.0001**	**0.75 (0.69-0.80), p < 0.0001**	**0.71 (0.66-0.77), p < 0.0001**	**1.11 (1.04-1.19), p < 0.05**
RBBB Phenogroup 6	**1.29 (1.13-1.47), p < 0.01**	**1.35 (1.24-1.47), p < 0.0001**	**1.53 (1.39-1.69), p < 0.0001**	**1.19 (1.09-1.31), p < 0.001**	**1.25 (1.15-1.37), p < 0.0001**	**1.28 (1.17-1.39), p < 0.0001**
AF atrial fibrillation; ASCVD atherosclerotic cardiovascular disease; BIDMC Beth Israel Deaconess Medical Center; EF ≤ 50% impaired left ventricular function (ejection fraction < 50%); HF heart failure; IVCD intraventricular conduction delay; LBBB left bundle branch block; MI myocardial infarction; NSIVCD non-specific IVCD; RBBB right bundle branch block; VA ventricular arrythmia.						

Table S7

Adjusted hazard ratios from multivariate time-to-event models evaluating associations between phenogroup assignments and incident cardiovascular disease and mortality outcomes in the BIDMC broad QRS DDRTree population. Cox proportional hazards (HR) are reported for the fatal outcomes while Fine-gray sub-distribution hazards (SHR) are reported for the non-fatal outcomes, to account for the competing risk of death. Exponentiated hazards ratios, 95% confidence intervals and associated adjusted p-values (corrected for multiple testing, p-value x 9) are presented for each phenogroup, against baseline comparator RBBB phenogroup 4, adjusted for covariates. Covariates included age, sex, ECG measurements (heart rate, QRS duration, QTc interval) and type of QRS morphology (left or right bundle branch block (LBBB/RBBB) or non-specific intraventricular conduction delay (NSIVCD)). All-cause mortality was treated as the competing event for other incident outcomes.

Phenogroup	VA: SHR (95% CI, p-value)	HF: SHR (95% CI, p-value)	AF: SHR (95% CI, p-value)	EF ≤ 50: SHR (95% CI, p-value)	ASCVD: SHR (95% CI, p-value)	MI: SHR (95% CI, p-value)	CV death: HR (95%CI, p-value)	CHB: SHR (95% CI, p-value)	Mortality: HR (95%CI, p-value)
LBBB Phenogroup 1	**1.41 (1.13-1.76), p < 0.05**	**1.36 (1.16-1.58), p < 0.001**	0.95 (0.80-1.13), 5.22	0.66 (0.44-0.98), 0.37	1.14 (0.94-1.38), 1.62	1.17 (0.98-1.41), 0.81	**1.36 (1.15-1.61), p < 0.01**	**1.64 (1.33-2.03), p < 0.0001**	1.14 (1.03-1.27), 0.11
LBBB-NSIVCD Phenogroup 2	**1.61 (1.32-1.98), p < 0.0001**	**1.32 (1.16-1.51), p < 0.001**	0.96 (0.81-1.12), 5.22	1.08 (0.75-1.54), 6.21	1.25 (1.06-1.48), 0.07	1.14 (0.96-1.35), 1.17	**1.25 (1.07-1.47), p < 0.05**	**1.94 (1.61-2.33), p < 0.0001**	1.11 (1.01-1.22), 0.27
IVCD Phenogroup 3	**1.47 (1.23-**	1.11 (0.99-1.25), 0.63	1.16 (1.01-	1.10 (0.80-	1.17 (1.01-1.35), 0.32	1.05 (0.91-	**1.27 (1.11-1.45), p < 0.01**	**1.26 (1.07-1.49), p < 0.05**	1.10 (1.01-1.19), 0.25

	**1.76), p < 0.001**		1.33), 0.32	1.51), 4.95		1.22), 4.50			
RBBB Phenogroup 5	**0.71 (0.61-0.82), p < 0.0001**	0.91 (0.84-0.99), 0.20	**0.84 (0.76-0.92), p < 0.01**	0.80 (0.63-1.01), 0.53	1.01 (0.91-1.12), 7.83	**0.82 (0.74-0.92), p < 0.01**	**0.75 (0.68-0.83), p < 0.0001**	**0.74 (0.66-0.84), p < 0.0001**	**0.83 (0.78-0.89), p < 0.0001**
RBBB Phenogroup 6	**1.31 (1.12-1.53), p < 0.01**	1.05 (0.95-1.16), 3.24	1.06 (0.95-1.19), 2.70	1.01 (0.76-1.35), 8.37	**1.24 (1.09-1.42), p < 0.05**	0.98 (0.86-1.12), 7.11	**1.46 (1.30-1.63), p < 0.0001**	1.17 (1.02-1.34), 0.21	**1.24 (1.16-1.33), p < 0.0001**

AF atrial fibrillation; ASCVD atherosclerotic cardiovascular disease; CHB complete heart block; CV cardiovascular; EF ≤ 50% impaired left ventricular function (ejection fraction < 50%); HF heart failure; IVCD intraventricular conduction delay; LBBB left bundle branch block; NSIVCD non-specific IVCD; RBBB right bundle branch block; VA ventricular arrythmia.

Table S8

Adjusted odds ratios from multivariate logistic models evaluating the association between phenogroup assignments and prevalent cardiovascular disease outcomes in the BIDMC broad QRS DDRTree population. Exponentiated odds ratios, 95% confidence intervals and associated p-values are presented for each phenogroup, against the baseline comparator RBBB phenogroup 4, adjusted for covariates. Covariates included age, sex, ECG measurements (heart rate, QRS duration, QTc interval). This analysis was also performed as a sensitivity analysis excluding QRS morphology to assess its impact on the associations.

	**Exponentiated Odds Ratio (95% confidence intervals) p-value**					
**Phenogroup**	**VA**	**HF**	**EF ≤ 50%**	**ASCVD**	**MI**	**AF**
LBBB Phenogroup 1	1.03 (0.90-1.18), 0.64	**2.12 (1.95-2.30), p < 0.0001**	**3.15 (2.87-3.46), p < 0.0001**	**1.12 (1.03-1.22), p < 0.01**	1.04 (0.95-1.14), 0.36	0.96 (0.89-1.05), 0.38
LBBB-NSIVCD Phenogroup 2	**1.48 (1.32-1.66), p < 0.0001**	**2.08 (1.93-2.24), p < 0.0001**	**3.11 (2.86-3.40), p < 0.0001**	**1.08 (1.00-1.17), p < 0.05**	**1.22 (1.13-1.32), p < 0.0001**	**1.39 (1.29-1.49), p < 0.0001**
IVCD Phenogroup 3	**2.05 (1.82-2.31), p < 0.0001**	**1.73 (1.60-1.88), p < 0.0001**	**2.51 (2.29-2.76), p < 0.0001**	0.99 (0.91-1.08), 0.86	**1.26 (1.16-1.37), p < 0.0001**	**2.05 (1.89-2.22), p < 0.0001**
RBBB Phenogroup 5	**0.88 (0.79-0.99), p < 0.05**	**0.73 (0.68-0.78), p < 0.0001**	**0.66 (0.60-0.72), p < 0.0001**	**0.75 (0.70-0.80), p < 0.0001**	**0.71 (0.65-0.76), p < 0.0001**	**1.09 (1.02-1.17), p < 0.05**
RBBB Phenogroup 6	**1.35 (1.19-1.54), p < 0.0001**	**1.38 (1.27-1.51), p < 0.0001**	**1.57 (1.42-1.74), p < 0.0001**	**1.19 (1.09-1.30), p < 0.001**	**1.26 (1.15-1.38), p < 0.0001**	**1.31 (1.21-1.43), p < 0.0001**

AF atrial fibrillation; ASCVD atherosclerotic cardiovascular disease; BIDMC Beth Israel Deaconess Medical Center; EF ≤ 50% impaired left ventricular function (ejection fraction < 50%); HF heart failure; IVCD intraventricular conduction delay; LBBB left bundle branch block; MI myocardial infarction; NSIVCD non-specific IVCD; RBBB right bundle branch block; VA ventricular arrythmia.

Table S9

Adjusted hazard ratios from multivariate time-to-event models evaluating associations between phenogroup assignments and incident cardiovascular disease and mortality outcomes in the BIDMC broad QRS DDRTree population. Cox proportional hazards (HR) are reported for the fatal outcomes while Fine-gray sub-distribution hazards (SHR) are reported for the non-fatal outcomes, to account for the competing risk of death. Exponentiated hazards ratios, 95% confidence intervals and associated p-values are presented for each phenogroup, against baseline comparator RBBB phenogroup 4, adjusted for covariates. Covariates included age, sex, ECG measurements (heart rate, QRS duration, QTc interval). All-cause mortality was treated as the competing event for other incident outcomes. This analysis was also performed as a sensitivity analysis excluding QRS morphology to assess its impact on the associations. All-cause mortality was treated as the competing event for other incident outcomes.

**Phenogroup**	**VA: SHR (95% CI, p-value)**	**HF: SHR (95% CI, p-value)**	**AF: SHR (95% CI, p-value)**	**EF ≤ 50: SHR (95% CI, p-value)**	**ASCVD: SHR (95% CI, p-value)**	**MI: SHR (95% CI, p-value)**	**CV death: HR (95%CI, p-value)**	**CHB: SHR (95% CI, p-value)**	**Mortality: HR (95%CI, p-value)**
LBBB Phenogroup 1	**1.59 (1.37-1.84), p < 0.0001**	**1.60 (1.45-1.77), p < 0.0001**	1.04 (0.94-1.17), 0.43	**1.55 (1.19-2.02), p < 0.01**	**1.23 (1.08-1.40), p < 0.01**	**1.24 (1.10-1.40), p < 0.001**	**1.21 (1.08-1.35), p < 0.001**	1.09 (0.95-1.25), 0.21	**1.08 (1.00-1.15), p < 0.05**
LBBB-NSIVCD Phenogroup 2	**1.82 (1.59-2.08), p < 0.0001**	**1.55 (1.42-1.69), p < 0.0001**	1.07 (0.97-1.18), 0.18	**2.29 (1.83-2.87), p < 0.0001**	**1.29 (1.15-1.45), p < 0.0001**	**1.17 (1.05-1.31), p < 0.01**	1.10 (0.99-1.22), 0.08	**1.41 (1.26-1.59), p < 0.0001**	1.06 (0.99-1.12), 0.08
IVCD Phenogroup 3	**1.63 (1.41-1.88), p < 0.0001**	**1.24 (1.13-1.36), p < 0.0001**	**1.33 (1.19-1.47), p**	**1.58 (1.24-2.03), p < 0.001**	1.11 (0.98-1.25), 0.11	1.04 (0.92-1.17), 0.58	**1.12 (1.00-1.25), p < 0.05**	1.13 (0.99-1.28), 0.07	1.07 (1.00-1.14), 0.06

			**< 0.0001**						
RBBB Phenogroup 5	**0.71 (0.61-0.82), p < 0.0001**	**0.91 (0.84-0.98), p < 0.05**	**0.83 (0.76-0.92), p < 0.001**	0.80 (0.63-1.01), 0.06	1.02 (0.91-1.13), 0.78	**0.82 (0.74-0.92), p < 0.001**	**0.75 (0.68-0.84), p < 0.0001**	**0.74 (0.66-0.84), p < 0.0001**	**0.83 (0.78-0.89), p < 0.0001**
RBBB Phenogroup 6	**1.32 (1.12-1.54), p < 0.001**	1.05 (0.95-1.17), 0.31	1.08 (0.96-1.21), 0.22	1.03 (0.77-1.37), 0.84	**1.23 (1.08-1.40), p < 0.01**	0.98 (0.86-1.12), 0.77	**1.45 (1.29-1.61), p < 0.0001**	**1.16 (1.02-1.33), p < 0.05**	**1.24 (1.16-1.33), p < 0.0001**
AF atrial fibrillation; ASCVD atherosclerotic cardiovascular disease; CHB complete heart block; CV cardiovascular; EF ≤ 50% impaired left ventricular function (ejection fraction < 50%); HF heart failure; IVCD intraventricular conduction delay; LBBB left bundle branch block; NSIVCD non-specific IVCD; RBBB right bundle branch block; VA ventricular arrythmia.									

Table S10

Frequency of incident events across the UKB (UK Biobank) cohort.

Outcome	Event Rate (%)
Myocardial infarction	1.75
Heart failure	2.77
Atrial fibrillation	3.16
All-cause mortality	1.94

Table S11

Multivariate regressions of phenogroup assignments against prevalent outcomes within the UK Biobank (UKB) population.

The exponentiated odds ratio estimates, 95% confidence intervals and associated p-values are presented for each phenogroup against the prevalent outcomes, adjusted for covariates, against the baseline comparator RBBB phenogroup 4. Covariates included age, sex and type of QRS morphology (left or right bundle branch block (LBBB/RBBB) or non-specific intraventricular conduction delay (NSIVCD)).

	**Exponentiated Odds Ratio (95% confidence intervals) p-value**		
	**Myocardial Infarction**	**Heart Failure**	**Atrial Fibrillation**
**LBBB Phenogroup 1**	0.77 (0.27-2.24), 0.64	**8.36 (1.58-52.21), p < 0.05**	0.83 (0.29-2.37), 0.72
**LBBB-NSIVCD Phenogroup 2**	1.20 (0.45-3.22), 0.71	**10.31 (2.05-61.25), p < 0.01**	1.56 (0.61-3.99), 0.36
**IVCD Phenogroup 3**	1.29 (0.55-2.98), 0.55	**6.65 (1.56-34.04), p < 0.05**	0.85 (0.34-2.03), 0.73
**RBBB Phenogroup 5**	**0.53 (0.30-0.95), p < 0.05**	1.40 (0.46- 6.07), 0.60	1.04 (0.63-1.80), 0.87
**RBBB Phenogroup 6**	1.68 (0.81-3.42), 0.15	2.89 (0.63-14.89), 0.17	1.31 (0.60-2.76), 0.48
LBBB left bundle branch block; NSIVCD non-specific intraventricular conduction delay; RBBB right bundle branch block			

Table S12

Multivariate regressions of phenogroup assignments against incident outcomes within the UK Biobank (UKB) population. The exponentiated hazards ratio estimates, 95% confidence intervals and associated p-values are presented for each phenogroup against the incident outcomes, adjusted for covariates, against the baseline comparator RBBB phenogroup 4. Cox proportional hazards (HR) are reported for the mortality endpoint while Fine-gray sub-distribution hazards (SHR) are reported for the remaining outcomes, to account for the competing risk of death. Covariates included age, sex and type of QRS morphology (left or right bundle branch block (LBBB/RBBB) or non-specific intraventricular conduction delay (NSIVCD)). All-cause mortality was treated as the competing event for other incident outcomes.

	**MI: SHR (95% CI, p-value)**	**HF: SHR (95% CI, p-value)**	**AF: SHR (95% CI, p-value)**	**Mortality: HR (95%CI, p-value)**
**LBBB Phenogroup 1**	0.56 (0.08- 3.91), 0.56	4.05 (0.89-18.35), 0.07	1.20 (0.32-4.46), 0.79	2.22 (0.25-19.97), 0.48
**LBBB-NSIVCD Phenogroup 2**	0.61 (0.09- 4.20), 0.62	**4.03 (1.06-15.39), p < 0.05**	2.21 (0.75-6.50), 0.15	2.92 (0.36-23.42), 0.31
**IVCD Phenogroup 3**	1.08 (0.16- 7.30), 0.94	1.45 (0.30- 7.09), 0.64	0.56 (0.18-1.77), 0.32	1.88 (0.34-10.49), 0.47
**RBBB Phenogroup 5**	1.98 (0.45- 8.60), 0.36	0.39 (0.12- 1.28), 0.12	**0.44 (0.22-0.92), p < 0.05**	1.20 (0.40- 3.58), 0.74
**RBBB Phenogroup 6**	3.90 (0.76-20.04), 0.10	1.71 (0.45- 6.44), 0.43	1.18 (0.48-2.91), 0.71	1.87 (0.47- 7.51), 0.38

AF atrial fibrillation; HF heart failure; MI myocardial infarction; LBBB left bundle branch block; NSIVCD non-specific intraventricular conduction delay; RBBB right bundle branch block

Table S13

Multivariate regressions of phenogroup assignments against echocardiography measures in the BIDMC population. The beta estimates, 95% confidence intervals and associated p-values for the phenogroups against echocardiography measures (LVEF, LVEDD and LVESD) adjusted for the covariates are shown, using RBBB phenogroup 4 as the baseline comparator. Covariates included age, sex and type of QRS morphology (left or right bundle branch block (LBBB/RBBB) or non-specific intraventricular conduction delay (NSIVCD)).

BIDMC Beth Israel Deaconess Medical Center.

	Beta estimate (95% confidence intervals)		
**Phenogroup**	**LVEF (%)**	**LVEDD (mm)**	**LVESD (mm)**
Higher risk LBBB Phenogroup 1	**-2.10 (-3.79 to -0.41), p < 0.05**	**-0.98 (-1.90 to -0.05), p < 0.05**	0.47 (-0.83 to 1.77), 0.48
Higher risk LBBB-NSIVCD Phenogroup 2	**-3.07 (-4.65 to -1.50), p < 0.001**	0.04 (-0.82 to 0.90), 0.93	**1.46 (0.25 to 2.66), p < 0.05**
Higher risk IVCD Phenogroup 3	**-4.12 (-5.54 to -2.71), p < 0.0001**	**1.90 (1.12 to 2.67), p < 0.0001**	**2.88 (1.81 to 3.95), p < 0.0001**
Lower risk RBBB Phenogroup 5	**1.54 (0.50 to 2.59), p < 0.01**	-0.13 (-0.69 to 0.43), 0.65	-0.12 (-0.88 to 0.63), 0.75
Higher risk RBBB Phenogroup 6	**-2.41 (-3.63 to -1.20), p < 0.0001**	0.56 (-0.10 to 1.22), 0.10	0.50 (-0.40 to 1.40), 0.27

LBBB left bundle branch block; LVEF left ventricular ejection fraction; LVEDD left ventricular end-diastolic diameter; LVESD left ventricular end-systolic diameter; NSIVCD non-specific intraventricular conduction delay; RBBB right bundle branch block.

Table S14

Investigating CRT response along LBBB-dominant phenogroups 1 and 2 in the BIDMC population. Exponentiated odds ratios for CRT response endpoints (LVEF 10% increase and LVESD 15% decrease) regressed against position along the branch (moving from the start of a branch towards its periphery). Both estimates from unadjusted and adjusted analyses are shown with the respective 95% confidence intervals and associated p-values. The covariates adjusted for included age, sex and QRS duration. *BIDMC Beth Israel Deaconess Medical Center.*

	**Higher risk LBBB phenogroup 1**		**Higher risk LBBB-NSIVCD phenogroup 2**	
**Position along branch**	**LVEF 10% increase**	**LVESD 15% decrease**	**LVEF 10% increase**	**LVESD 15% decrease**
Unadjusted	1.58 (0.39-6.37) 0.52	0.85 (0.12-6.22) 0.87	**7.66 (1.78-32.89) p < 0.01**	**26.43 (1.43-489.03) p < 0.05**
Adjusted	1.28 (0.30-5.51) 0.74	3.15 (0.27-36.77) 0.36	**5.41 (1.13-26.01) p < 0.05**	7.74 (0.35-170.47) 0.19
LBBB left bundle branch block; LVEF left ventricular ejection fraction; LVESD left ventricular end-systolic diameter; NSIVCD non-specific intraventricular conduction delay; RBBB right bundle branch block.				

Figure S1

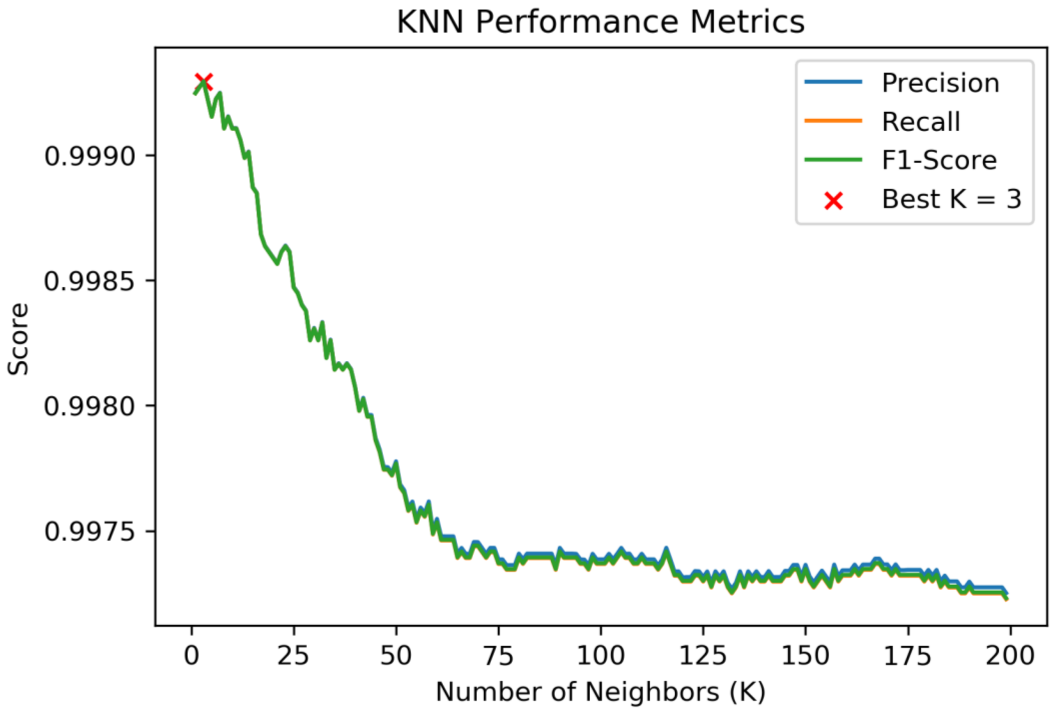

Performance of the K-Nearest Neighbors (KNN) classification model obtained on the internal hold-out test set from the broad QRS BIDMC population for predicting phenogroup assignment in the UK Biobank cohort. K of 3 was found to achieve the best performance across all the metrics.

BIDMC Beth Medical Isreal Deaconess Centre.

Figure S2

A

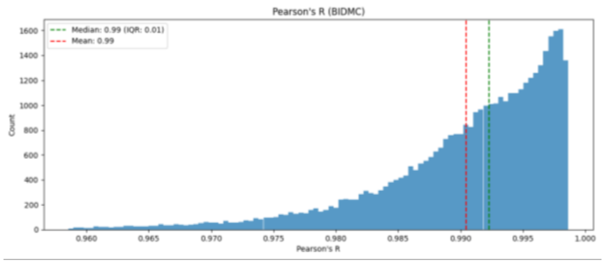

B

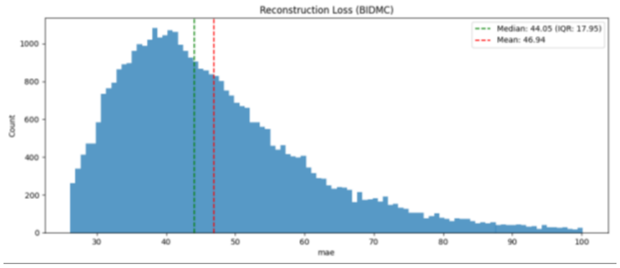

C

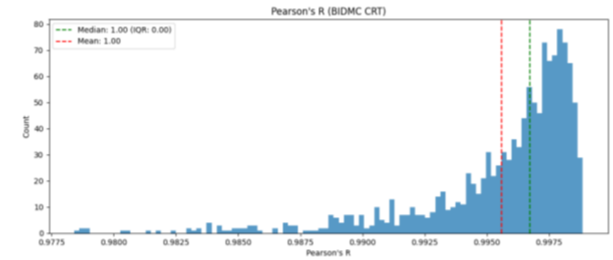

D

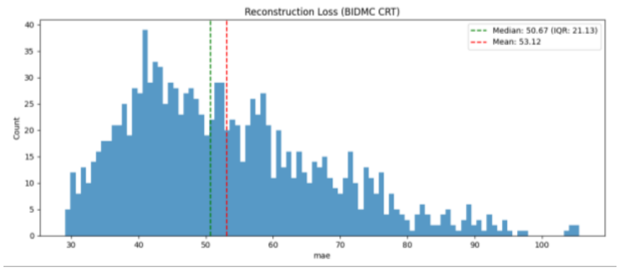

Evaluating performance of median beat reconstruction by the VAE model in the broad QRS BIDMC population. A) Pearson's R and B) Reconstruction loss for the non-CRT data subset of BIDMC. C) Pearson's R and D) Reconstruction loss for the BIDMC-CRT data subset. *BIDMC Beth Israel Deaconess Medical Centre; CRT cardiac resynchronisation therapy; VAE variational autoencoder.*

Figure S3

A

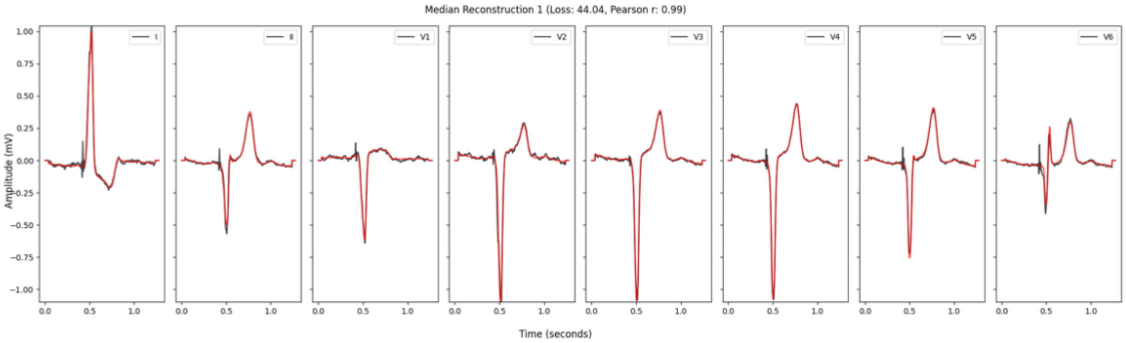

B

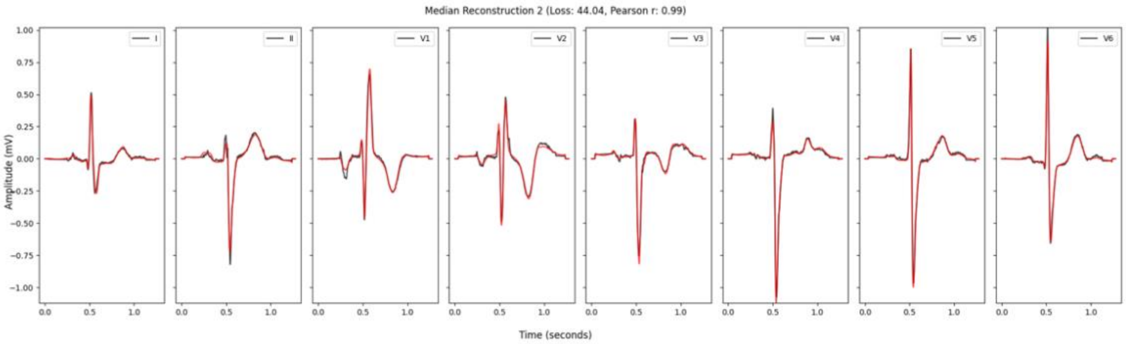

C

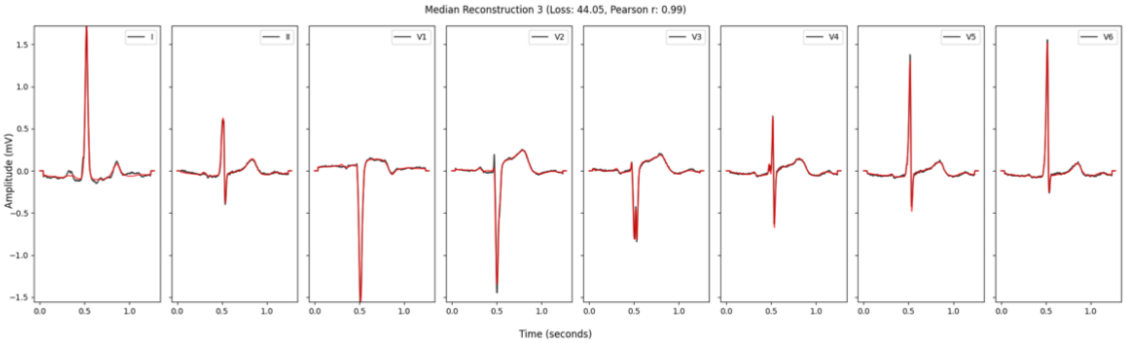

D

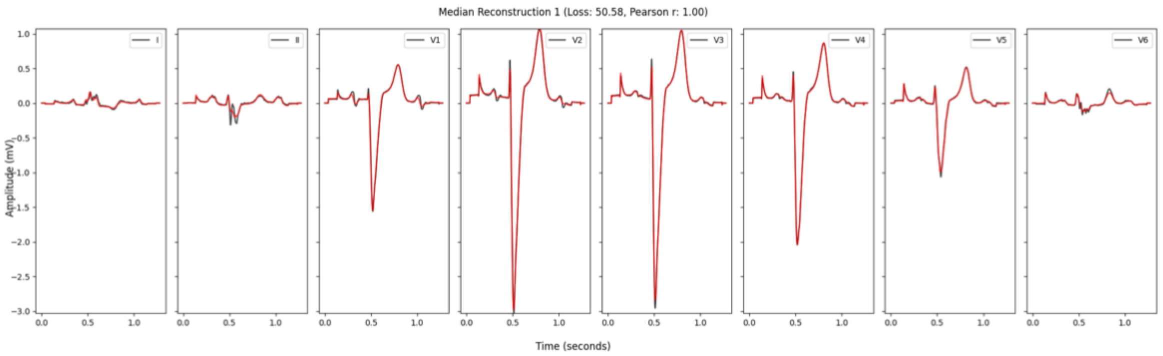

E

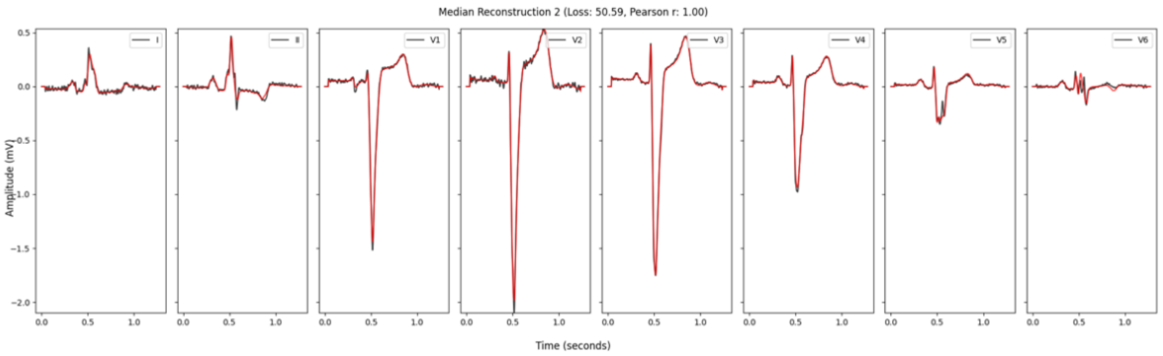

F

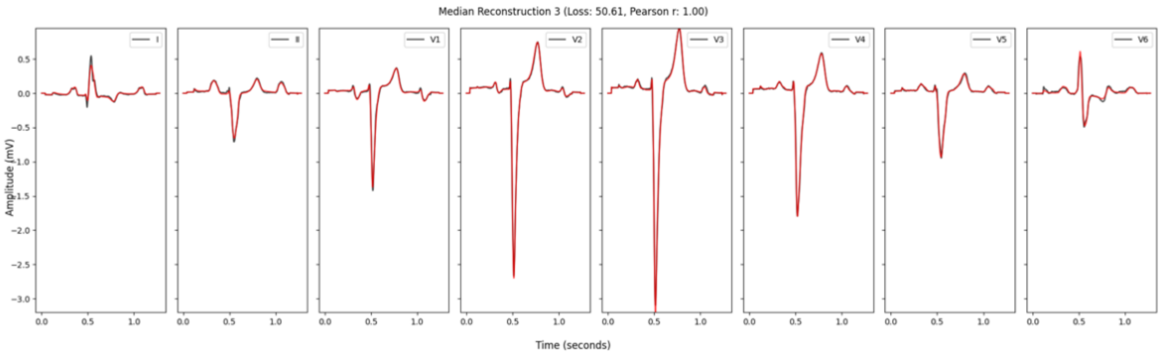

Examples of median beat reconstructions from the broad QRS BIDMC population using the VAE model. A-C show reconstructed median beats from the non-CRT subset, while D-F show reconstructed median beats from the BIDMC-CRT subset.

BIDMC Beth Israel Deaconess Medical Centre; CRT cardiac resynchronisation therapy; VAE variational autoencoder.

Figure S4

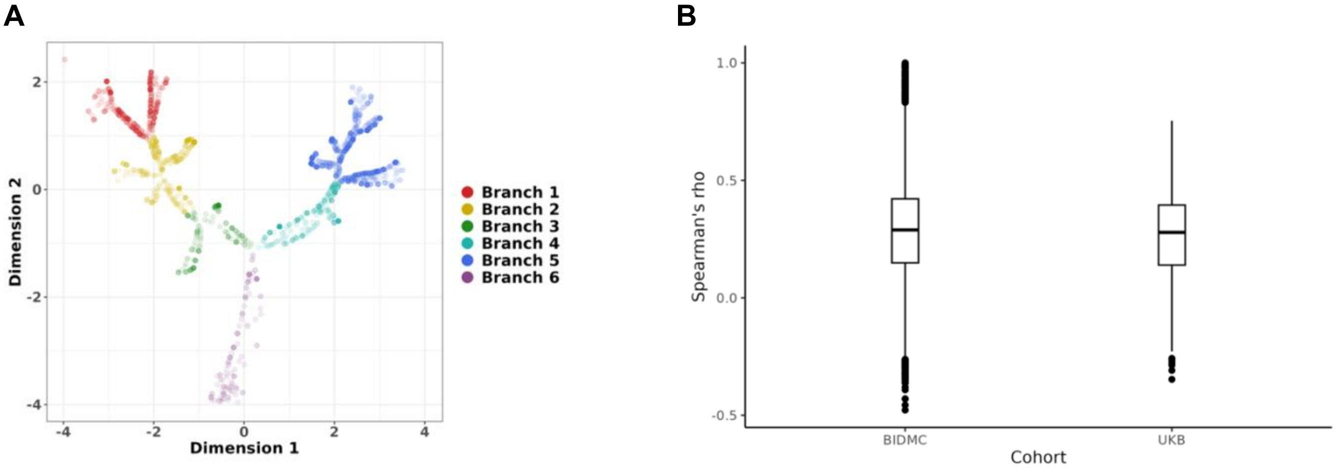

Projecting the UKB broad QRS population onto the BIDMC DDRTree. A) shows the predicted tree variables (branch assignments and the dimension coordinates) for the UKB population from supervised models trained on the BIDMC DDRTree data. B) shows the similarity between the nearest tree points between the 2 cohorts. Spearman's correlation coefficient between the latent features of the nearest BIDMC points in the tree follow the same distribution as the nearest UK Biobank and BIDMC points.

BIDMC Beth Israel Deaconess Medical Centre; DDRTree dimensionality reduction via learning a tree, UKB UK Biobank.

Figure S5

A

Phenogroup	Lambda.1se	Alpha
1	0.00126	0.10
2	0.00696	0.10
3	0.00636	0.55
4	0.00197	1.00
5	0.000757	0.10
6	0.00117	0.55
Lambda.1se lambda value at one standard error from the minimum cross-validation error		

B

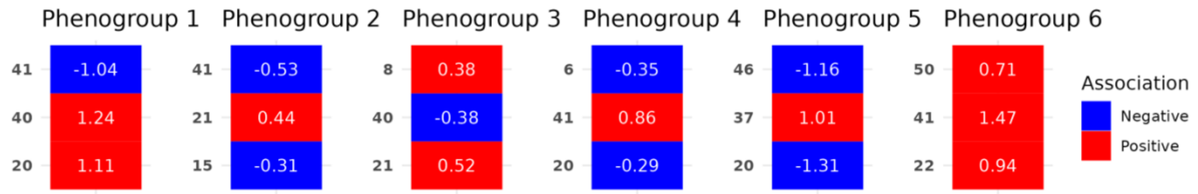

C

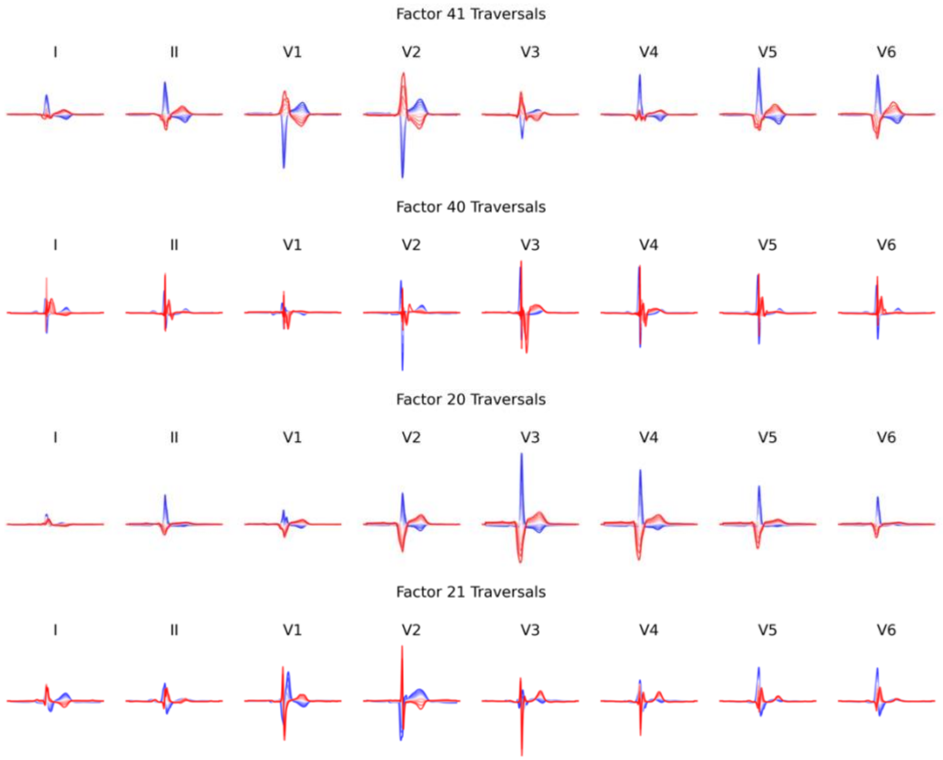

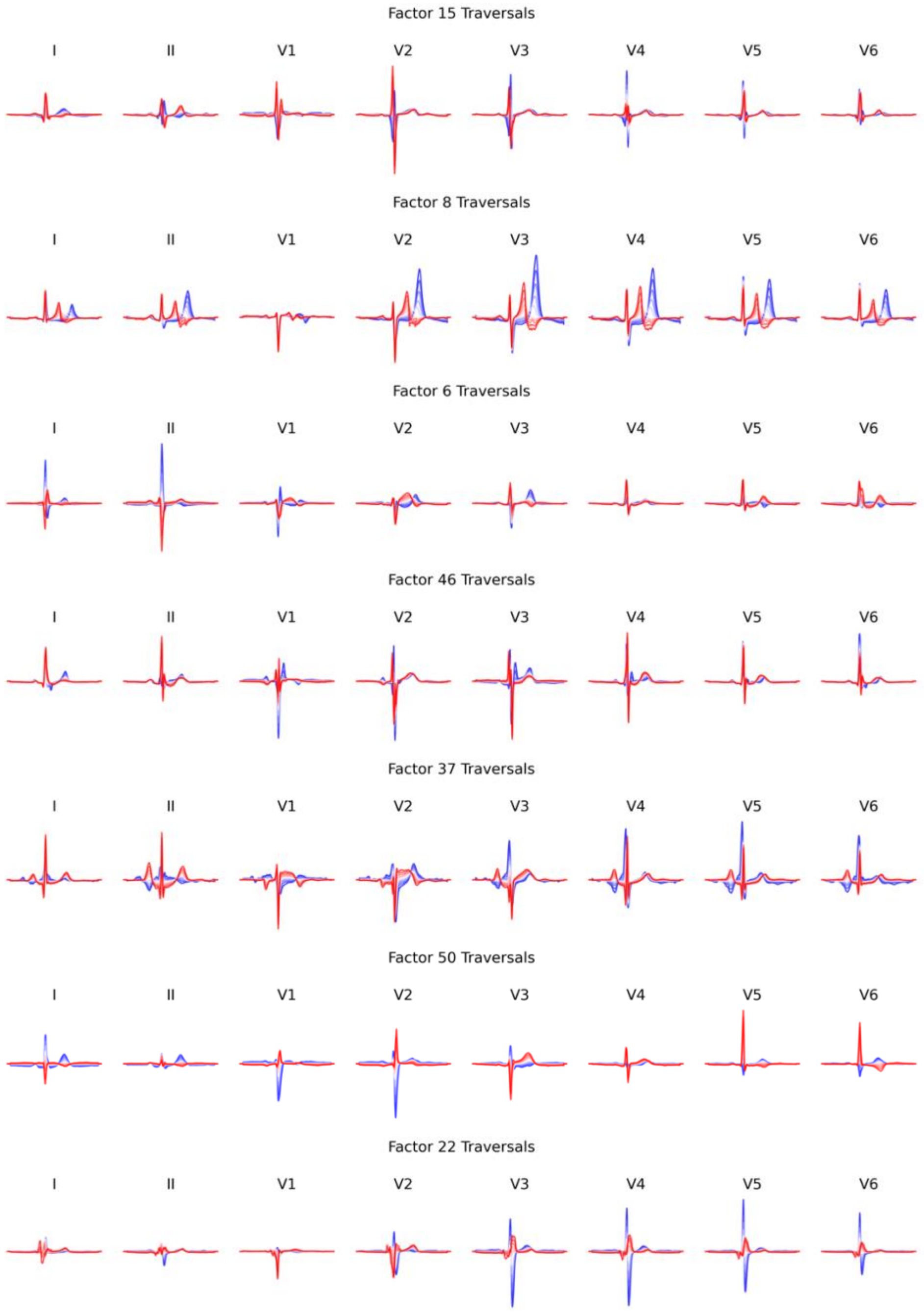

Explainability using latent ECG features across the DDRTree derived phenogroups in the BIDMC population. A) Tuned lambda (1 Standard Error value) and alpha values for each phenogroup derived from the regularised logistic regression. The lambda values represent the optimal regularisation strength for each phenogroup, while the alpha values indicate the balance between Lasso (L1) and Ridge (L2) penalties in the Elastic Net regularisation. These values were determined through cross-validation to select the best-fit parameters for each phenogroup.

Panel B) Top 3 latent features for each phenogroup assignment and associated adjusted log-odds estimates. The log-odds estimates reflect the strength and direction of the association between phenogroups and the latent features, adjusted for all other features. These estimates are obtained after applying regularisation to address multi-collinearity among the features. Panel C) Visualising the latent transversals for the selected latent features across the 8 ECG leads. *BIDMC Beth Israel Deaconess Medical Centre; DDRTree dimensionality reduction via learning a tree.*

Figure S6

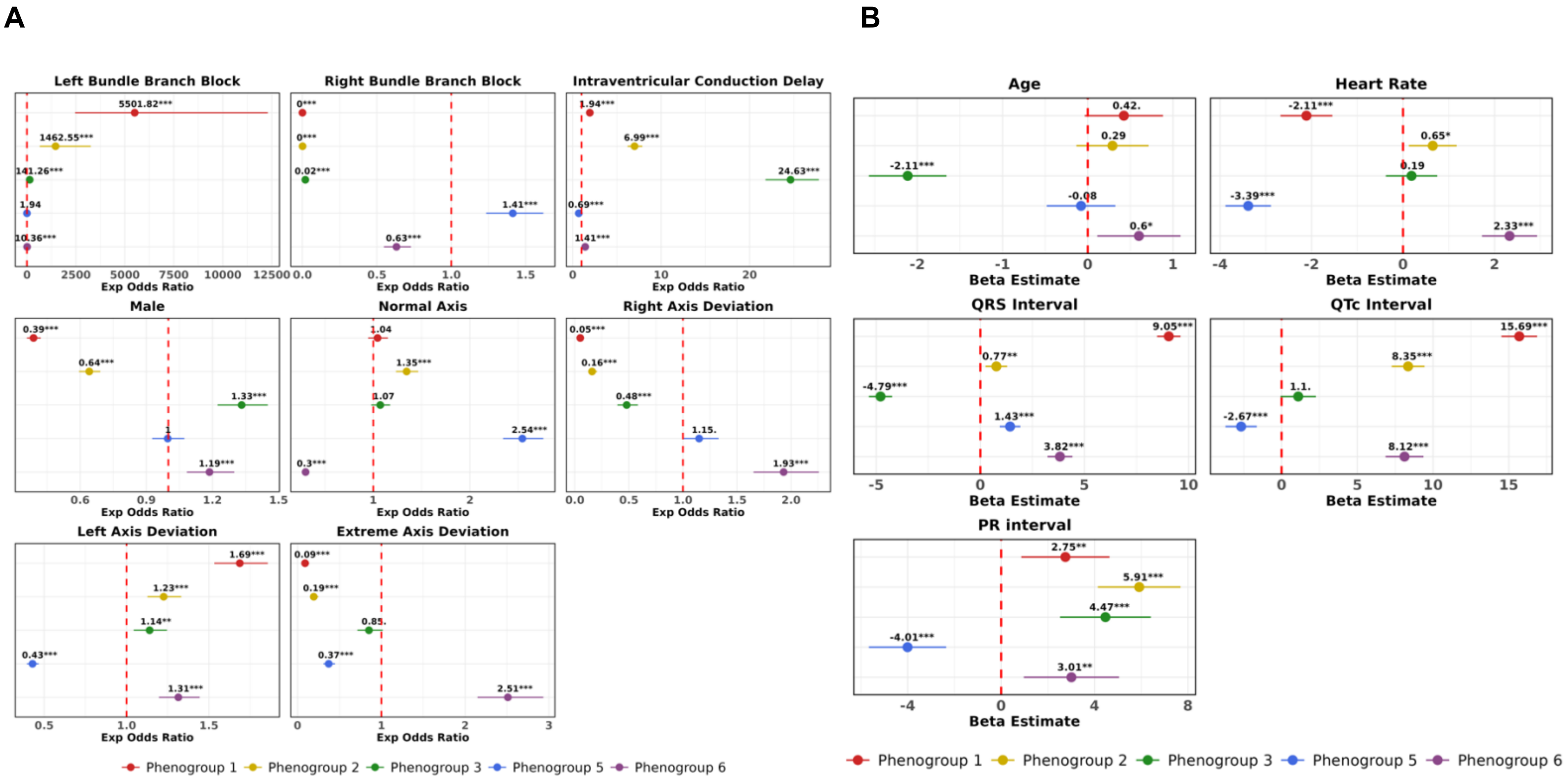

Univariate regression estimates of baseline variables against broad QRS phenogroup assignments in BIDMC. Panel A) Forest plots showing the exponentiated odds ratios for each phenogroup assignment against binary baseline variables using phenogroup 4 as the baseline comparator. The red line refers to an exponentiated odds ratio of 1, indicating non-significance. Panel B) Forest plots showing the beta (β) estimates for each phenogroup assignment against continuous baseline variables using phenogroup 4 as the baseline comparator. The red line refers to an adjusted β estimate of 0, indicating non-significance.

Figure S7

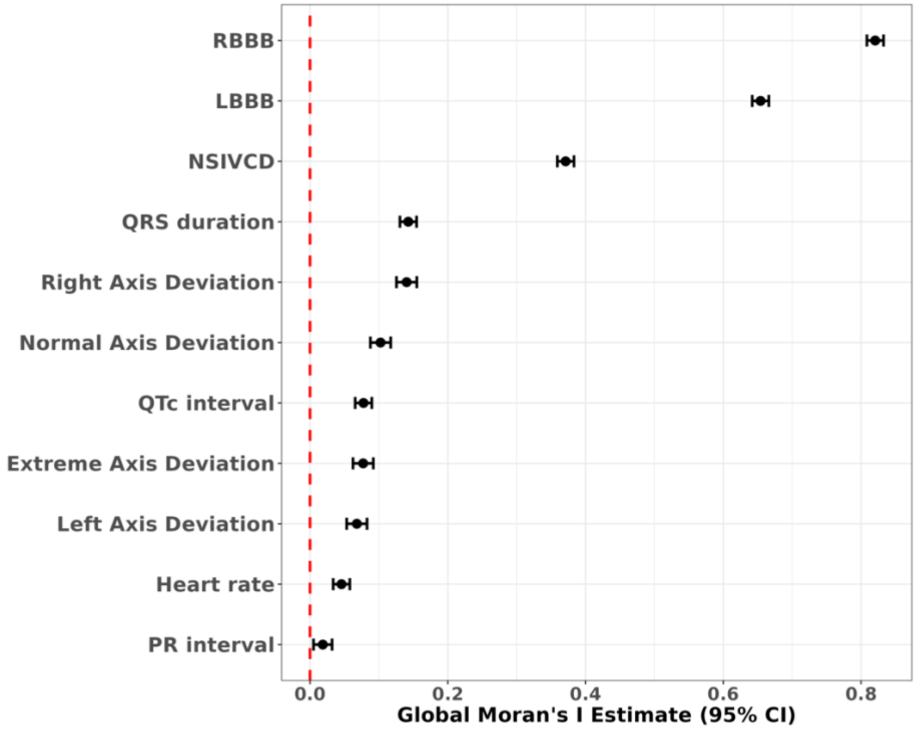

Spatial autocorrelation patterns of baseline and electrophysiological variables across the broad QRS BIDMC DDRTree. The dotted red line represents a Global Moran's I estimate of 0, indicating no spatial autocorrelation. All variable estimates presented were associated with a p-value < 0.01.

BIDMC Beth Medical Isreal Deaconess Centre; LBBB left bundle branch block; NSIVCD non-specific intraventricular conduction delay; RBBB right bundle branch block.

Figure S8

A

B

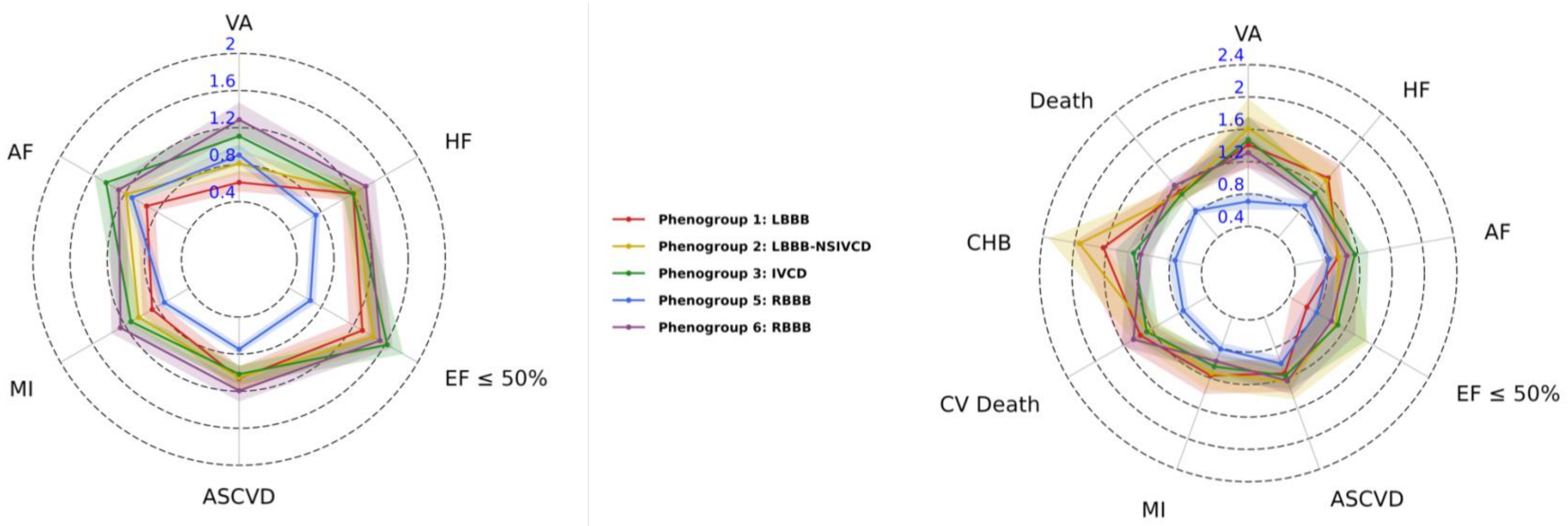

Radar plots showing the associations of broad QRS BIDMC DDRTree phenogroups with cardiovascular comorbidities and mortality outcomes. A) shows the adjusted odds ratios and 95% confidence intervals for each phenogroup for prevalent diseases, compared to baseline phenogroup 4. B) shows the adjusted hazards ratios and 95% confidence intervals for each phenogroups for incident diseases, compared to baseline phenogroup 4. Cox proportional hazards are reported for the fatal outcomes while Fine-gray sub-distribution hazards are reported for the non-fatal outcomes, to account for the competing risk of death. All estimates presented were adjusted for covariates including age, sex, ECG measurements (heart rate, QRS duration and QTc interval) and type of QRS morphology (left or right bundle branch block (LBBB/RBBB) and non-specific intraventricular conduction delay (NSIVCD)). Death was treated as the competing event for other incident outcomes.

AF atrial fibrillation; ASCVD atherosclerotic cardiovascular disease; BIDMC Beth Israel Deaconess Medical Center; CHB complete heart block; CV death cardiovascular death; DDRTree dimensionality reduction via learning a tree; EF ≤ 50% impaired left ventricular function (ejection fraction < 50%); HF heart failure; MI myocardial infarction; VA ventricular arrythmia.

Figure S9

A

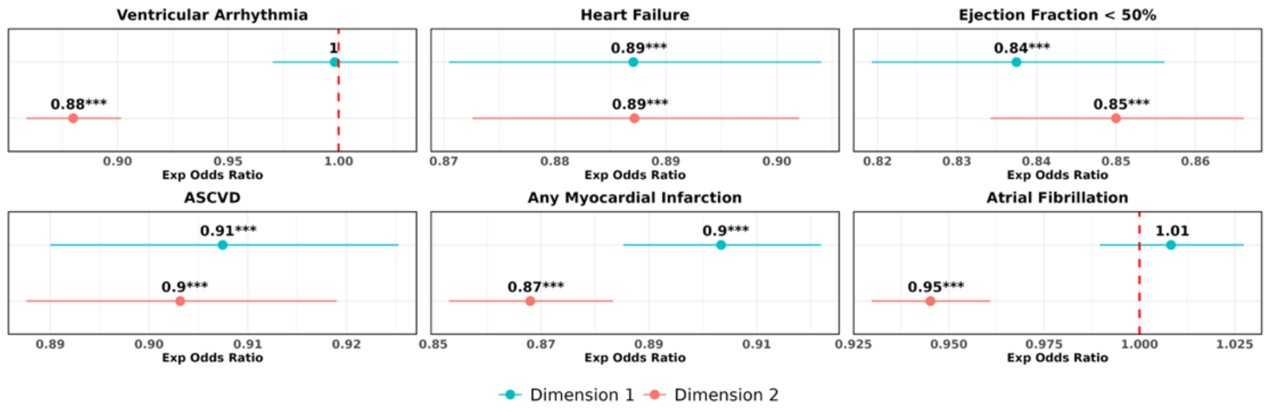

B

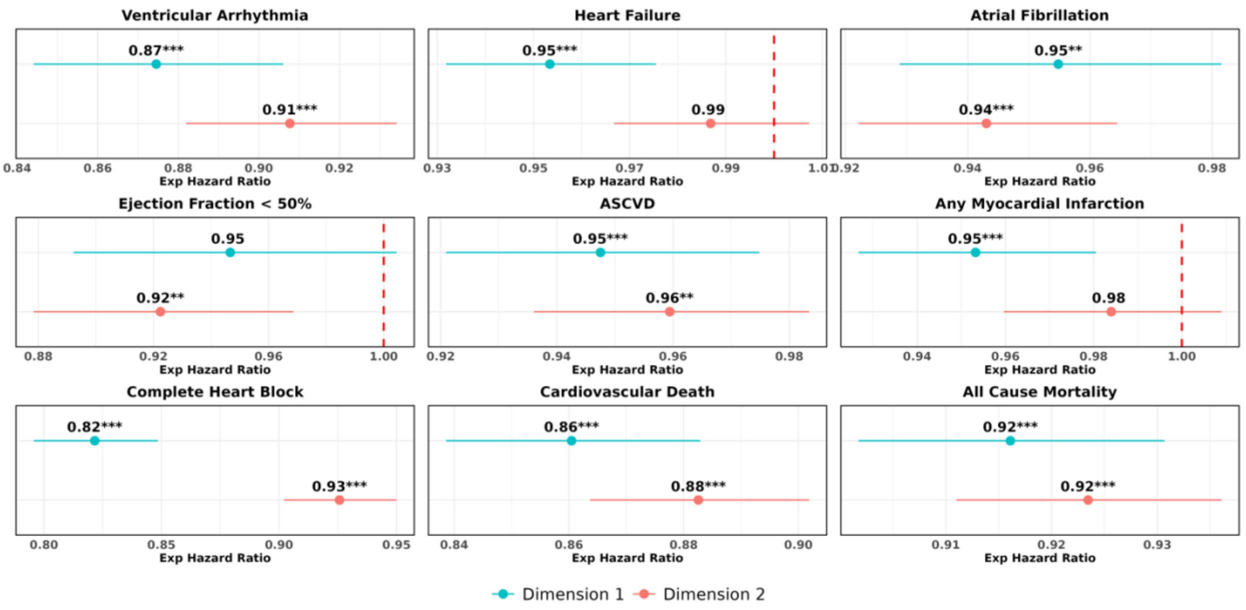

Multivariate regressions of tree dimensions (dimensions 1 and 2) against prevalent outcomes and time-to-event outcomes for the BIDMC cohort. Panel A) Forest plots showing the exponentiated odds ratios (OR) of tree variables against prevalent diseases, adjusted for covariates. Panel B) Forest plots showing the exponentiated hazard ratios (HR) of tree variables against incident disease outcomes, adjusted for covariates. Cox proportional hazards are

reported for the fatal outcomes while Fine-gray sub-distribution hazards are reported for the non-fatal outcomes, to account for the competing risk of death. The red line refers to an exponentiated OR/HR of 1, indicating non-significance. All regression models presented were adjusted for age, sex, heart rate, QRS, PR and QTc interval and type of QRS morphology - right or left bundle branch block, non-specific intraventricular conduction delay.

ASCVD atherosclerotic cardiovascular disease; BIDMC Beth Medical Isreal Deaconess Centre; EF 50% impaired ventricular function (ejection fraction ≤ 50%).

Figure S10

A

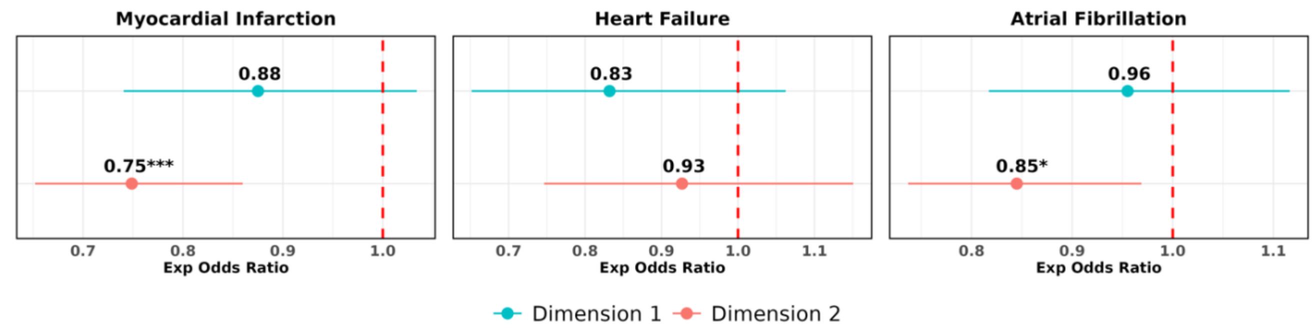

B

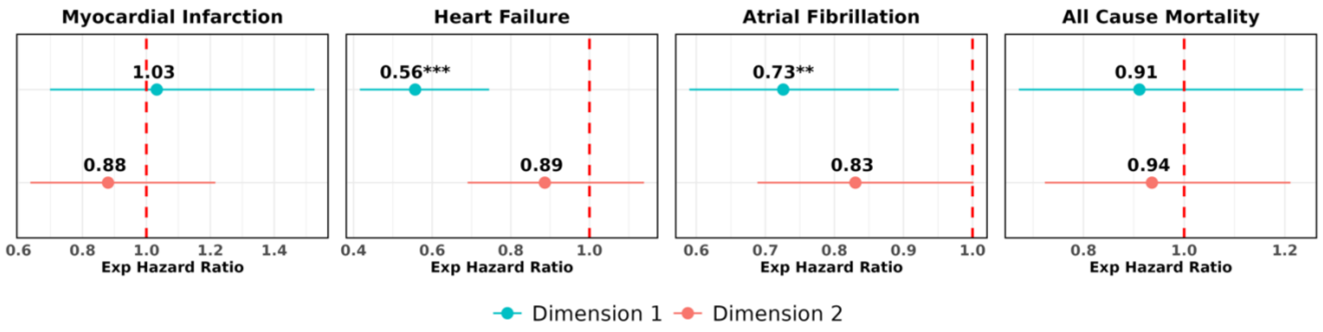

Multivariate regressions of tree dimensions (dimensions 1 and 2) against prevalent outcomes and time-to-event outcomes for the UK Biobank cohort. Panel A) Forest plots showing the adjusted odds ratios (aOR) of tree variables against prevalent diseases, adjusted for covariates. Panel B) Forest plots showing the adjusted hazard ratios (aHR) of tree variables against incident disease outcomes, adjusting for covariates. Cox proportional hazards are reported for the all-cause mortality endpoint while Fine-gray sub-distribution hazards are reported for the remaining non-fatal outcomes, to account for the competing risk of death. The red line refers to an exponentiated OR/SHR of 1, indicating non-significance. All regression models presented were adjusted for age, sex and type of QRS morphology - right or left bundle branch block, non-specific intraventricular conduction delay.

Figure S11

A

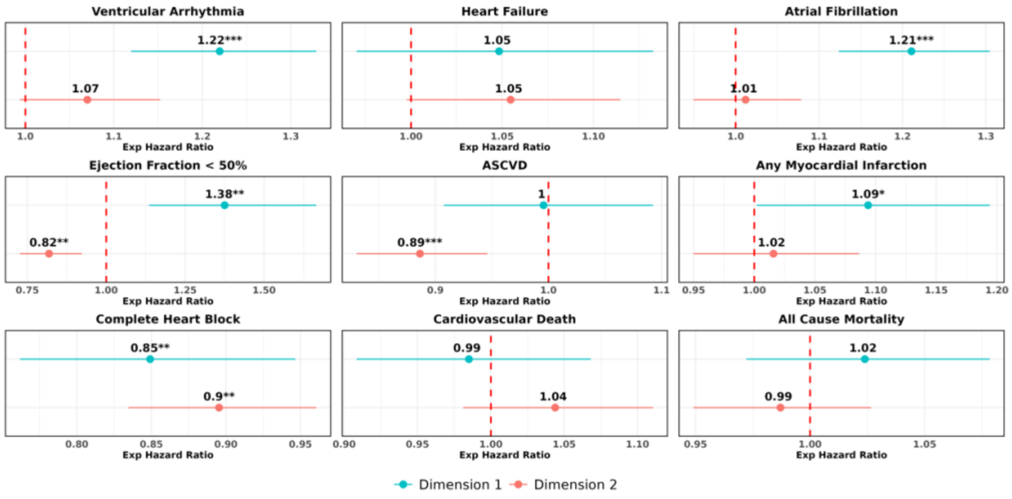

B

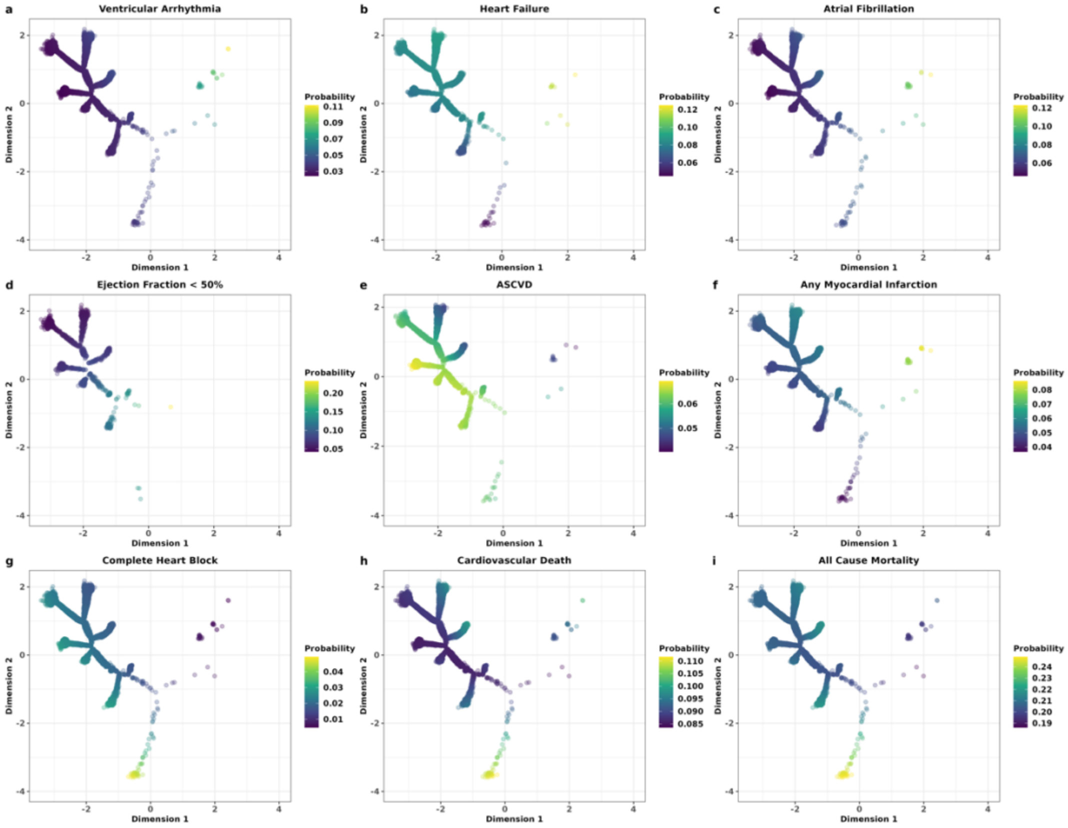

Assessing trends of future risk of disease in LBBB ECGs from the broad QRS BIDMC population. Panel A) Forest plots showing the adjusted hazard ratios (aHR) of tree dimensions

(dimensions 1 and 2) against incident disease outcomes, adjusting for covariates. Cox proportional hazards are reported for the fatal outcomes while Fine-gray sub-distribution hazards are reported for the non-fatal outcomes, to account for the competing risk of death. The red line refers to an adjusted aHR of 1, indicating non-significance. All regression models presented were adjusted for age, sex and ECG measurements (QRS, PR and QTc intervals). Panel B) Predicted probability of disease for LBBB ECGs across incident outcomes overlaid across the tree. These were estimated using Cox proportional hazards models for the fatal outcomes and Fine-gray sub-distribution models for the non-fatal outcomes, using tree dimensions and pseudotime (position within the tree, relative to the tree core) as model inputs. *ASCVD atherosclerotic cardiovascular disease; BIDMC Beth Medical Isreal Deaconess Centre; EF 50% impaired ventricular function (ejection fraction ≤ 50%).*

Figure S12

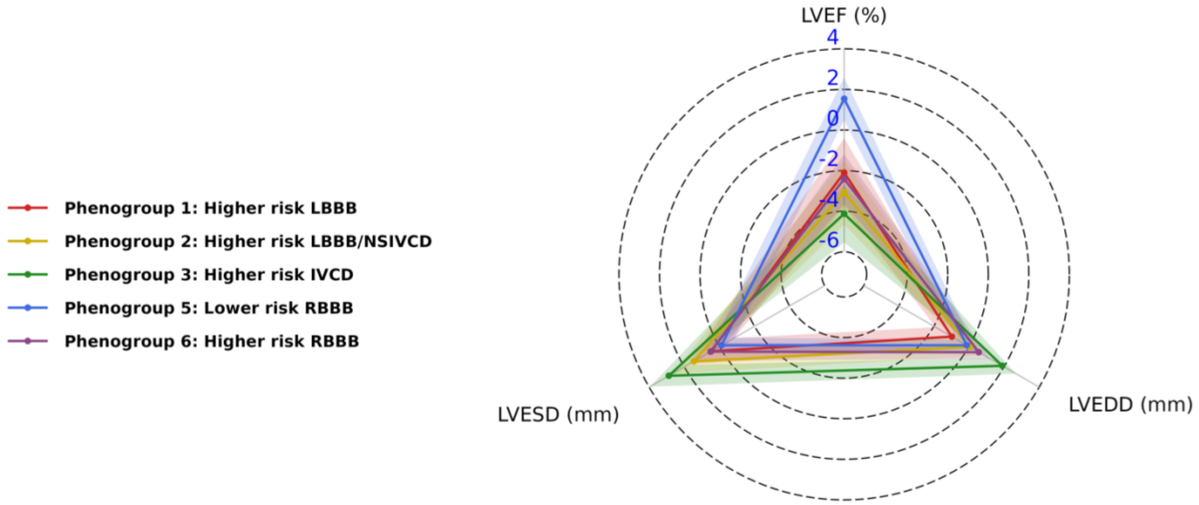

Radar plots showing the associations of broad QRS BIDMC DDRTree phenogroups with echocardiography measures. Adjusted beta estimates and 95% confidence intervals are plotted for each phenogroup with LVEF, LVEDD and LVESD compared to baseline phenogroup 4. All estimates presented were adjusted for covariates including age, sex, ECG measurements (heart rate, QRS duration and QTc interval) and type of QRS morphology (left or right bundle branch block (LBBB/RBBB) and non-specific intraventricular conduction delay (NSIVCD)). *BIDMC Beth Medical Isreal Deaconess Centre; DDRTree dimensionality reduction via learning a tree; LVEF left ventricular ejection fraction; LVEDD left ventricular end-systolic diameter; LVESD left ventricular end-systolic diameter.*

Figure S13

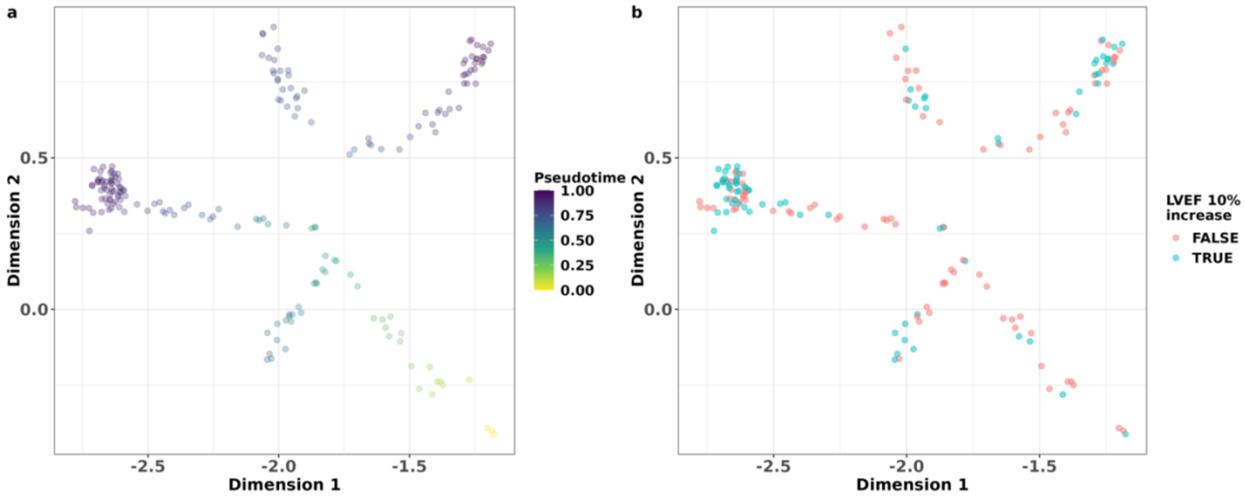

Visualising CRT response along higher risk LBBB-NSIVCD phenogroup 2 in the BIDMC cohort. A) shows the position of ECGs within LBBB-NSIVCD phenogroup 2 with increasing pseudotime indicating moving towards the branch peripheries. B) shows the CRT response endpoint of LVEF 10% increase overlaid across ECGs within phenogroup 2.

BIDMC Beth Isreal Deaconess Medical Centre; CRT cardiac resynchronisation therapy; LBBB left bundle branch block; LVEF left ventricular conduction delay; NSIVCD non-specific intraventricular conduction delay.

Supplementary references

48. Burgess, C. P. *et al.* Understanding disentangling in β-VAE. Preprint at https://arxiv.org/abs/1804.03599 (2018).
